# Genetic factors inherited from both diploid parents interact to affect genome stability and fertility in resynthesized allotetraploid *Brassica napus*

**DOI:** 10.1093/g3journal/jkad136

**Published:** 2023-06-14

**Authors:** Elizabeth Ihien Katche, Antje Schierholt, Sarah-Veronica Schiessl, Fei He, Zhenling Lv, Jacqueline Batley, Heiko C Becker, Annaliese S Mason

**Affiliations:** Plant Breeding Department, University of Bonn, Bonn 53115, Germany; Department of Plant Breeding, Justus Liebig University, Giessen 35392, Germany; Department of Crop Sciences, Division of Plant Breeding Methodology, Georg-August University Göttingen, Göttingen 37073, Germany; Department of Plant Breeding, Justus Liebig University, Giessen 35392, Germany; Department of Botany and Molecular Evolution, Senckenberg Research Institute and Natural History Museum Frankfurt, Frankfurt am Main D-60325, Germany; Plant Breeding Department, University of Bonn, Bonn 53115, Germany; Plant Breeding Department, University of Bonn, Bonn 53115, Germany; Department of Plant Breeding, Justus Liebig University, Giessen 35392, Germany; School of Biological Sciences, University of Western Australia, Perth, WA 6009, Australia; Department of Crop Sciences, Division of Plant Breeding Methodology, Georg-August University Göttingen, Göttingen 37073, Germany; Plant Breeding Department, University of Bonn, Bonn 53115, Germany; Department of Plant Breeding, Justus Liebig University, Giessen 35392, Germany

**Keywords:** copy number variation, fertility, genome stability, meiosis, resynthesized *Brassica napus*, single nucleotide polymorphism

## Abstract

Established allopolyploids are known to be genomically stable and fertile. However, in contrast, most newly resynthesized allopolyploids are infertile and meiotically unstable. Identifying the genetic factors responsible for genome stability in newly formed allopolyploid is key to understanding how 2 genomes come together to form a species. One hypothesis is that established allopolyploids may have inherited specific alleles from their diploid progenitors which conferred meiotic stability. Resynthesized *Brassica napus* lines are often unstable and infertile, unlike *B. napus* cultivars. We tested this hypothesis by characterizing 41 resynthesized *B. napus* lines produced by crosses between 8 *Brassica rapa* and 8 *Brassica oleracea* lines for copy number variation resulting from nonhomologous recombination events and fertility. We resequenced 8 *B. rapa* and 5 *B. oleracea* parent accessions and analyzed 19 resynthesized lines for allelic variation in a list of meiosis gene homologs. SNP genotyping was performed using the Illumina Infinium *Brassica* 60K array for 3 individuals per line. Self-pollinated seed set and genome stability (number of copy number variants) were significantly affected by the interaction between both *B. rapa* and *B. oleracea* parental genotypes. We identified 13 putative meiosis gene candidates which were significantly associated with frequency of copy number variants and which contained putatively harmful mutations in meiosis gene haplotypes for further investigation. Our results support the hypothesis that allelic variants inherited from parental genotypes affect genome stability and fertility in resynthesized rapeseed.

## Introduction

Polyploidy is the heritable condition of possessing more than 2 sets of chromosomes (Comai 2005). The extra set/s of chromosomes may originate from the same individual or from within the same species, which is referred to as autopolyploidy, or from hybridization between 2 different species, known as allopolyploidy (Otto 2007). Polyploidy confers a number of evolutionary advantages (Soltis and Soltis 2000), including increased potential for heterosis due to the contribution of additional gene copies to a trait, and genetic redundancy which allows additional gene copies to take on new functions without loss of established and required functions in the organism (reviewed by Comai 2005). Polyploids do however face significant challenges to establishment, including initial self-propagation, and reproductive isolation from and competition with parental progenitor species (reviewed by Mable 2013 and Shimizu 2022). One of the most significant barriers to polyploid establishment is thought to be regulation of meiosis (Pelé *et al.* 2018). In newly formed polyploids, cell machinery must adapt to the presence of extra chromosomes: in autopolyploids via enforcement of a single crossover per chromosome pair per meiosis and in allopolyploids via strict segregation of homologous chromosomes belonging to each of the progenitor genomes (prevention of nonhomologous chromosome pairing) (reviewed by Bomblies 2023).

How the process of meiotic adaptation to polyploidy occurs is still relatively unknown across most taxa, although insights into the genetic mechanisms involved have now been gained in several species (reviewed by Bomblies 2023). In bread wheat (*Triticum aestivum*), prevention of homoeologous crossovers is known to be regulated by the *Ph1* locus (Griffiths *et al.* 2006), which contains a duplicated and diverged copy of meiosis gene *ZIP4* (*TaZIP4-B2*), a gene which is essential for homologous crossover formation as well as synapsis in wheat (Martín *et al.* 2021). In autopolyploid *Arabidopsis arenosa*, 8 meiosis genes were initially identified as under selective sweeps related to adaptation to polyploidy (Yant *et al.* 2013); of these, polyploid-adapted alleles of meiotic chromosome axis formation genes *ASY1* and *ASY3* (Morgan *et al.* 2020) as well as interacting chromatin condensation and axis recruitment gene *REC8* (Morgan *et al.* 2022) have so far been shown to act in stabilizing polyploid meiosis.

The *Brassica* genus is 1 of 51 genera in the tribe Brassiceae belonging to the crucifer family (Brassicaceae) and is the most economically important genus within this tribe (Rakow 2004). It is an interesting model for allopolyploid formation in agricultural crops as 6 agriculturally significant species share a genomic interrelationship (U 1935). *Brassica napus* (genome A_n_A_n_C_n_C_n_) was spontaneously formed by recent allopolyploidy between ancestors of *Brassica oleracea* (Mediterranean cabbage, genome C_o_C_o_) and *Brassica rapa* (Asian cabbage or turnip, genome A_r_A_r_) in the last 7,500 years and is thought to be polyphyletic in origin (Allender and King 2010; Chalhoub *et al.* 2014). Therefore, the *Brassica* genus and most especially *B. napus* are increasingly receiving attention as a model for regulation of meiosis in a young polyploid crop species (Mason and Snowdon 2016).

To date, some progress has been made in identifying potential causes for the stabilization of meiosis in allopolyploid *B. napus*, which is thought to be under quantitative genetic control, similar to Brassicaceae relative *Arabidopsis* species (Liu *et al.* 2006; Higgins *et al.* 2021; Morgan *et al.* 2022). Although no molecular characterization of gene candidates has been carried out as in *Arabidopsis* and bread wheat, quantitative trait loci mapping approaches have revealed at least 1 major-effect locus (contributing 32–58% to homoeologous recombination frequency), for which a differentially expressed meiosis gene candidate *RPA1C* was identified (Higgins *et al.* 2021). Recent work with mutation (knockout) lines has also suggested that many different pathways to meiotic stabilization may be possible within *Brassica* allopolyploids (Gonzalo *et al.* 2019; Gonzalo 2022). As well, retention of meiosis gene copies following earlier polyploidization events in the *Brassica* lineage (Lloyd *et al.* 2014) and the presence of preexisting meiotic gene variants in diploid species which may contribute to enhanced stabilization of meiosis in the polyploid have been suggested as possible mechanisms for meiotic stabilization (Cifuentes *et al.* 2010).

Synthetic polyploids can be produced through genome doubling of diploid plants or hybrids via methods such as chemical treatment with colchicine (Spoelhof *et al.* 2017). However, most synthetic polyploids remain largely unstable in terms of meiosis and genome inheritance (reviewed by Pelé *et al.* 2018). Meiotic aberrations are common in newly formed autopolyploid and allopolyploid plants, which negatively affects their fertility and early demographic success (Ramsey and Schemske 2002; Gaeta and Pires 2010). Synthetic allopolyploids lack the phenotypic and genomic stability of established allopolyploids (Soltis and Soltis 1995; Pikaard 1999; Comai *et al.* 2000). Abnormal phenotypes and frequent failure of pollen and embryo development (Schranz and Osborn 2000; Comai *et al.* 2003) as well as widespread changes in gene expression have been observed in other synthetic allopolyploids (Kashkush *et al.* 2002). Although not all newly resynthesized allopolyploids are genomically unstable (Comai *et al.* 2000; Wang *et al.* 2006; Novikova *et al.* 2017; Chen *et al.* 2020), most are, including synthetic *Brassica* allotetraploids (Song *et al.* 1995), and this has been attributed to abnormal meiosis (Szadkowski *et al.* 2010). This meiotic instability involves homoeologous pairing or interactions between the closely related A and C genome chromosomes during meiosis (Comai *et al.* 2000; Nicolas *et al.* 2007, 2012; Leflon *et al.* 2010). Although several studies have produced and investigated synthetic *Brassica* (e.g. Abel *et al.* 2005; Szadkowski *et al.* 2010; Mason *et al.* 2010; Girke *et al.* 2012; Jesske *et al.* 2013; Karim *et al.* 2014), almost all synthetic *Brassica* lines investigated so far appear to be meiotically unstable (Chen *et al.* 2011; Gaebelein and Mason 2018).

The question then is why the established *B. napus* species is stable and the resynthesized lines are unstable. One hypothesis is that *B. napus* may have gained genetic control via the inheritance of specific alleles from its diploid progenitor species, while a competing hypothesis suggests that mutations in the newly formed *B. napus* allopolyploid were selected on to restore meiotic stability (reviewed by Cifuentes *et al.* 2010). An increase in mutations as a result of interspecific hybridization (also known as “genomic shock”) has been established in several species (reviewed by Jackson and Chen 2010 and Soltis *et al.* 2016), famously maize (McClintock 1984), and nonhomologous translocations that have been observed in synthetic *B. napus* (Gaeta *et al.* 2007; Xiong *et al.* 2011; Samans *et al.* 2017) may also comprise a mechanism for novel mutations to restore meiotic function. The lack of observation of consistent translocations or other fixed genomic rearrangements in established *B. napus* relative to progenitor species *B. rapa* and *B. oleracea* (Chalhoub *et al.* 2014; Samans *et al.* 2017) fails to provide support for this hypothesis, although other types of as-yet-undetected mutations (e.g. transposable element-induced) in the newly formed resynthesized lines may also be responsible (Zou *et al.* 2011; Fu *et al.* 2016). These 2 hypotheses are also not exclusive, and recent reports in *Arabidopsis* have also suggested that evolution of meiotic stability may be a more gradual process, with polygenic selection for allelic variants, novel mutations, or regulations of gene expression that improve regularity of chromosome recombination and segregation and hence genome stability and fertility (Burns *et al.* 2021; Morgan *et al*. 2021).

A few studies have been conducted to explain genome instability in resynthesized *B. napus* allotetraploids (Gaeta *et al.* 2007; Szadkowski *et al.* 2010; Xiong *et al.* 2011), and recently several quantitative trait loci were identified to be present in natural *B. napus* that confer reduced homoeologous recombination rates (Higgins *et al.* 2021). However, no study to date has investigated multiple genotypes of resynthesized *B. napus* in order to test the idea that allelic variation inherited from the progenitor species conferred meiotic stability to natural *B. napus*. In this study, we aimed to test the hypothesis that allelic variants inherited from diploid progenitor species *B. rapa* and *B. oleracea* affect the frequency of homoeologous recombination in resynthesized *B. napus*, and hence, that inherited allelic variation may have conferred genomic stability to resynthesized *B. napus* lines.

## Materials and methods

### Description of plant material

The materials used in this study comprise resynthesized *B. napus* seeds derived from crosses between homozygous *B. rapa* and *B. oleracea* parents as described in Abel *et al*. (2005), where these are referred to as “spring-type domesticated lines.” The parental genotypes are either doubled haploid or highly inbred lines. C genome genotypes are either cauliflower (*B. oleracea* var. *botrytis*) or Chinese kale (*B. oleracea* var. *alboglabra*), and A genome genotypes are yellow sarson (*B. rapa* ssp*. trilocularis*), oilseed turnip (*B. rapa* ssp. *oleifera*, listed as *B. rapa* var. *rapa* in Abel *et al*. 2005), and Chinese cabbage (*B. rapa* ssp*. pekinensis*) (Supplementary File 1). Abel *et al*. (2005) produced seeds from 336 cross combinations between 21 *B. rapa* (maternal parent) and 16 *B. oleracea* lines including a core set of 64 cross combinations between 8 *B. rapa* and 8 *B. oleracea* lines. *B. rapa* was always the maternal parent in the crosses. Seeds from 41 resynthesized *B. napus* genotypes produced from crosses between 8 *B. rapa* (A4, A6, A7, A8, A9, A13, A16, and A19) and 8 *B. oleracea* parent genotypes (C34, C36, C37, C38, C42, C46, C47, and C49) via embryo rescue, chromosome doubling and self-pollination (S_1_ generation),and their *B. rapa* and *B. oleracea* parent genotypes were used in this study. Established *Brassica* cultivars were used as controls for fertility in our experiment: winter-type *B. napus* ‘Darmor’, spring-type *B. napus* ‘Argyle’, and semiwinter-type *B. napus* ‘Ningyou7', as well as *B. rapa* var. *oleifera* (unknown accession) and *B. oleracea* var. *botrytis* ‘NGB 1810.2'.

The resynthesized *B. napus* lines are represented by codes in the form “A1C1,” where “A1” is the *B. rapa* parent genotype and “C1” is the *B. oleracea* parent genotype. In the present study, 3 seeds from each of 41 resynthesized genotypes and cultivars of established *B. napus*, *B. rapa*, and *B. oleracea* used as controls were sown in quick pots and seedlings transferred to small pots without vernalization between September and November 2017 under glasshouse conditions at Justus Liebig University (JLU). Eight *B. rapa* parent genotypes (A4, A6, A7, A8, A9, A13, A16, and A19) as well as 6 *B. oleracea* lines (C34, C36, C37, C38, C46, and C47) used to produce the resynthesized *B. napus* lines were likewise sown on 2019 September 12 under the same glasshouse conditions at JLU as the resynthesized lines. All lines except C38 successfully germinated and produced plants. Three plant replicates from each genotype were then isolated in bags at flowering to ensure self-pollination.

### Assessment of purity in resynthesized *B. napus* lines

The purity of 41 resynthesized *B. napus* genotypes with SNP genotyping information was assessed. Eight parent *B. rapa*, 5 double haploid (DH) lines (A6, A7, A8, A9, and A13) and 3 inbred lines (A4, A16, and A19), as well as 8 *B. oleracea* genotypes, 5 DH lines (C34, C36, C37, C38, and C42) and 3 inbred lines (C46, C47, and C49), were used to produce the resynthesized lines (Abel *et al* 2005). Resynthesized lines produced by DH parental crosses between *B. rapa* and *B. oleracea* (AA × CC) are expected to be completely homozygous. Therefore, individuals of the same progeny sets having the same maternal *B. rapa* (AA) or paternal *B. oleracea* (CC) parents are expected to be nonsegregating. We assessed purity using 2 criteria: (1) the absence of segregation pattern among progeny sets and (2) the absence of continuous blocks of heterozygous (AB) calls across the A and the C genomes in all individuals.

### Fertility assessment in resynthesized *B. napus* lines

Three parameters were scored to describe fertility in resynthesized lines: relative pollen viability (as estimated by stainability), total number of seeds produced per plant, and number of seeds produced per 10 pods. Pollen viability was assessed for 2 freshly opened flowers per plant, and pollen grains stained with 1–2% acetocarmine solution (Leflon *et al.* 2006). At least 600 pollen grains per plant were counted and pollen viability was assessed using a light microscope (Leica DMR, Leica Microsystems), assuming darkly stained (red) pollen grains were viable and weakly stained or shrivelled pollen grains were nonviable. The total number of self-pollinated seeds produced per plant and the number of seeds produced per 10 pods were counted for each plant after harvesting. Individual plants were bagged after initiation of flowering using microperforated plastic bags to encourage self-pollination. The measure of total number of seeds per plant was collected as a very rough approximation of yield, while seeds per pod were assumed to relate better to meiotic process and % development of viable embryos.

### DNA extraction and genotyping using the Illumina Infinium *Brassica* 60K SNP array

Young leaf samples were collected in 2 mL microcentrifuge tubes at the 4–6 leaf stage of plant development. DNA was extracted for 41 resynthesized lines (123 plants) using the BioSprint 96 plant work station (Qiagen, Hilden, Germany) according to the manufacturer's instructions (http://qiagen.com/). SNP genotyping was carried out using the high-throughput Illumina Infinium 60K *Brassica* SNP array for the resynthesized lines. Hybridization protocols were performed according to the manufacturer’s instructions for all samples.

SNP data were analyzed, visualized, and exported into text files using Genome Studio v2.0.4 software (Illumina Inc., San Diego, CA, USA). All 52,149 SNPs were exported for the A and C genomes after application of the recommended “brassica60K” cluster file (Clarke *et al.* 2016). Top BLAST alignment hits for the SNP probes against the A and C genomes of the reference genome sequence of Darmor-*bzh* version 8.1 (Bayer *et al.* 2017) were used for genome position information. Hits to unplaced contigs were removed from further analyses. Data from samples of each *Brassica* species sourced from Mason *et al*. (2015) were used as controls for filtering SNPs. SNPs which mapped to the A genome but which amplified in *Brassica carinata* (BBCC) and *B. oleracea* (CC) genotype controls as well as SNPs which mapped to the C genome but amplified in *Brassica juncea* (AABB) and *B. rapa* (AA) genotype controls in >50% of the controls were filtered out. SNP markers with >50% heterozygous AB calls in all 5 *B. napus* homozygous control cultivars (Boomer, Monty_028DH, Surpass400_024DH, Trilogy, and Westar_10DH) as well as SNPs which showed >99% missing calls (NC) across all lines were removed. Further filtering steps included the removal of SNPs with ≥80% AB calls across individuals. After filtering, 21,938 SNPs were retained: 8,369 SNPs in the A genome and 13,569 SNPs in the C genome (Supplementary File 2). Genotype calls were then converted to homozygous/heterozygous calls (0 and 2 for homozygous and 1 for heterozygous) and incidence of missing calls represented by NA. Since the resynthesized lines were produced by chromosome doubling of parent F_1_ AC hybrids using colchicine, the allotetraploid hybrids are expected to be homozygous. After quality filtering of SNPs, we used the filtered SNPs to plot dendrograms separately for both the A and C genome parents (Supplementary Figs. 1 and 2).

### Detection of copy number variation in resynthesized *B. napus* lines

**Fig. 1.**
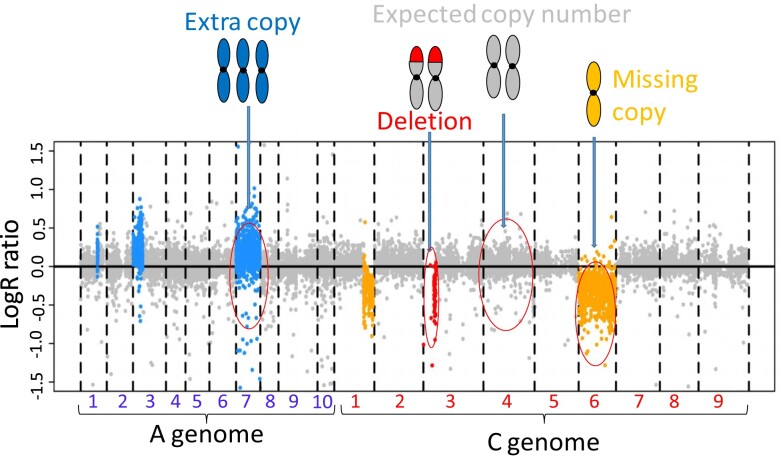
An example of copy number variant calling in resynthesized *B. napus* lines showing regions of higher copy number (blue: small regions on chromosomes A01 and A03 and all of chromosome A07), no copies/deletion (red: start of chromosome C03), expected copy (gray: majority of markers, e.g. chromosome C4), and single/reduced copy (orange: latter part of chromosome C1 and all of chromosome C6). Discrimination between 1 or 2 additional copies (3 or 4 copies total) was not possible using this method; these are hence referred to as “higher copy number” regions.

A copy number pipeline was developed in R (Schiessl *et al*., unpublished). The pipeline uses the log *R* ratios (Supplementary Files 3 and 4) to make plots for every individual line based on estimated cutoff values to score copy number variants. Log *R* ratios estimate relative fluorescence intensity for each SNP marker and are an output metric of Illumina GenomeStudio, the program used to call SNPs from raw data. For every SNP, we screened a diverse population representative for the diversity among natural *B. napus* (ASSYST) to get the expected log *R* ratio (Bus *et al.* 2012; Körber *et al.* 2012) distribution for this specific SNP. We then use quantiles to determine if the log *R* ratio of the SNP in our test population is unexpectedly low or high. The quantiles used for “deletion” were 10, for “missing copy” were 25, and for “extra copy” were 75: if SNP *x* in line *y* had a log *R* ratio value lower than the expected 10th quantile, SNP *x* was marked as a threshold SNP in line *y*. Windows with more than 5 threshold SNPs were kept and merged in case of physical overlap. The merged regions were reevaluated for the threshold SNP content, and regions with >50% threshold SNPs were retained. Regions with the same copy number variant (CNV) direction (extra copy/copies, or missing regions/deleted regions) that were very close together (<5 Mb) were merged (declared as deletion in the case that a deletion and a missing copy region were merged), and regions <2 Mb were also filtered out, so as not to overestimate CNV numbers based on noise in the data, especially since we only expect a limited number of nonhomologous recombination events per chromosome and hence smaller CNVs are less likely in this population (2 close-together nonhomologous recombination events are required for a small, nontelomeric CNV). Therefore, using the abovementioned criteria, we assessed CNVs in the resynthesized *B. napus* lines as deletion, missing/reduced copy, and extra/higher copy, with 2 copies as the expected copy number (Fig. 1). We also screened the log *R* ratio data of the resynthesized *B. napus* lines to check whether the same CNVs (inherited) are present in all progeny set derived from the *B. rapa* or *B. oleracea* parents.

### DNA extraction, sequencing, and sequence analysis of parental lines

DNA was extracted from young leaf samples of 8 *B. rapa* genotypes (A4, A6, A7, A8, A9, A13, A16, and A19) as well as 5 *B. oleracea* genotypes (C34, C36, C37, C46, and C47) using the Doyle and Doyle (1987) DNA extraction protocol. Illumina paired-end sequencing was performed at Novogene Company Limited, United Kingdom, on an Illumina HiSeq machine to produce reads of 150 bp length. Quality control was performed using FASTQC (https://www.bioinformatics.babraham.ac.uk/projects/fastqc/), and no further processing was found to be necessary. The parental reference genomes *B. rapa* cv. Chiifu version 3.0 (Zhang *et al.* 2018) and *B. oleracea* cv. JZS version 2.0 (Cai *et al.* 2020) were downloaded from the BRAD database (Chen *et al.* 2022). Reads were trimmed by removing low-quality reads and unpaired reads using TRIMMOMATIC version 0.39 (Bolger *et al.* 2014). Subsequently, reads were mapped to their respective reference genomes using bwa-mem (Li and Durbin 2010). Uniquely and high-quality mapping reads were selected by “samtools view -q 20” (-b: output is bam files, -q 20, mapping quality of phred score gives a 99% probability the mapping is correct) (Danecek *et al.* 2021). The depth of each base pair was obtained by “samtools depth -a” (-a all sites). SNP calling was performed using bcftools mpileup and filtered for a minimum quality of 30 and a minimum read depth of 10 using vcftools (Danecek *et al.* 2011), restricted to meiosis gene positions (Supplementary File 5). SNP annotation was performed using CooVar (Vergara *et al.* 2012).

### Detection of CNVs in parental *B. rapa* and *B. oleracea* lines

Since protein coding genes are more conserved and less repetitive than other parts of the genome, the detection of CNVs was carried out only for gene coding regions. The median sequencing depth of each gene (based on gene annotation) was calculated. These gene depths were then normalized by dividing by the mean depth of all genes. As our lines are not the same genotypes as the reference genome, there was probably some mapping bias. If all lines from 1 parent species showed low (<0.5-fold relative to mean coverage) or high (>1.5-fold relative to mean coverage) mapping rates, these genes were excluded from the analysis. To avoid uneven distribution of sequencing depth along the genome, a sliding window was calculated for the median depth of 40 genes. Relative read coverage for the median depth of 40 genes was carried out for each of the sequenced *B. rapa* and *B. oleracea* genotypes (Supplementary Figs. 3 and 4).

### Identification of meiosis gene candidates in resynthesized *B. napus*

A list of genes annotated as having functions in meiosis was established according to *Arabidopsis* gene annotation (TAIR) and additional published *Brassica* information (Lloyd *et al.* 2014). BLAST (Altschul *et al.* 1990) using an *E*-value cutoff of 10^−50^ was used to pull out meiosis gene copies based on sequence homology to *Arabidopsis* homologs in the *B. rapa* ‘Chiifu’ v2.5 (Cai *et al.* 2017) and *B. oleracea* ‘TO1000’ v. 2.1 (Parkin *et al.* 2014) reference genome assemblies. SNPs within these meiosis genes within the 5 *B. oleracea* genotypes (C34, C36, C37, C46, and C47) and 8 *B. rapa* genotypes (A4, A8, A9, A13, A16, and A19, excluding A6 and A7 which were found to be heterozygous) were analyzed using the following steps. Firstly, the SNP data of *B. oleracea/B. rapa* parents were read in. In the next step, all noninformative SNPs were removed: if 1 line had “missing information,” this SNP was excluded. Gene haplotypes were obtained from the SNP information. We then inferred the allelic state of the S_1_ resynthesized *B. napus* lines by combining the respective parents and subsequently matched these to the phenotype data (fertility, CNVs). Next, one-way ANOVA was used to test for significant differences between haplotypes and total CNVs as well as total seed set. The *P*-values were corrected using the false discovery rate (FDR) test. Subsequently, Fisher's exact test for count data was used to check for significant differences between putatively “stable” and “unstable” lines.

### Statistical analysis

Genotypic effects on fertility (self-pollinated seeds and seeds per 10 pods) and genome stability (as measured by number of CNVs) were tested for associations with alleles inherited from either *B. rapa* or *B. oleracea* parent or with the interaction between the 2 in the resynthesized lines. One-way ANOVA was used to test for significant differences in means followed by Tukey's Honest Significant Differences (HSD) test to assess differences between parent *B. rapa* and *B. oleracea* genotype groups using R v. 3.6.3 (The R Team for Statistical Computing).

## Results

### Purity of S_1_ resynthesized *B. napus* lines

SNP genotyping was carried out for all 41 resynthesized *B. napus* genotypes (123 individuals). All individual lines were homozygous and identical in allele inheritance to other individuals in the same progeny set, as expected. However, we observed unexpected differences in allele inheritance between progeny sets with the same parental lines C46, C49, and A19. For progeny sets sharing *B. oleracea* parent line C46, segregating regions were observed on chromosomes C06 (∼1.4 Mb) and C08 (2.4 Mb). Progeny sets with parent C49 also showed segregation on chromosomes C01 (∼1.1 Mb) and C04 (0.3 Mb). A 1 Mb region on chromosome A04 was also segregating between progeny sets A19C37, A19C47, and A19C49. Each of C46, C49, and A19 was homozygous inbred lines, rather than doubled haploid; this is likely the origin of the small regions of allelic heterozygosity hypothesized to be present in these progenitor genotypes.

We also observed large differences in A genome allele inheritance between progeny sets of resynthesized lines with *B. rapa* A6 and A7 as A genome parents: lines were homozygous within progeny sets but showed inheritance of different alleles from the A6 and A7 parents between progeny sets. Hence, parent genotypes A6 and A7 were likely actually heterozygous instead of homozygous as expected, explaining why the A genomes of different resynthesized genotype combinations with A6 and A7 *B. rapa* parents were not all the same (Supplementary Fig. 1). Consequently, we renamed all resynthesized *B. napus* lines which had A6 or A7 *B. rapa* parents with codes represented in the form “A6xC1” or “A7xCn,” where “x” constitutes a letter from a to f representing a genetically different parent *B. rapa* of that progeny set and “Cn” represents the *B. oleracea* parent genotype (C = *B. oleracea* and *n* = the genotype number). Other progeny sets with *B. rapa* parental genotypes A4, A8, A9, A13, and A16 as well as *B. oleracea* genotypes C34, C36, C37, C38, and C42 (Supplementary File 2) were completely homozygous, as no continuous blocks of heterozygosity and/or allele segregation between progeny sets were observed in these lines.

### Resynthesized *B. napus* lines show comparable fertility to parent *B. rapa* and *B. oleracea* genotypes

**Fig. 2.**
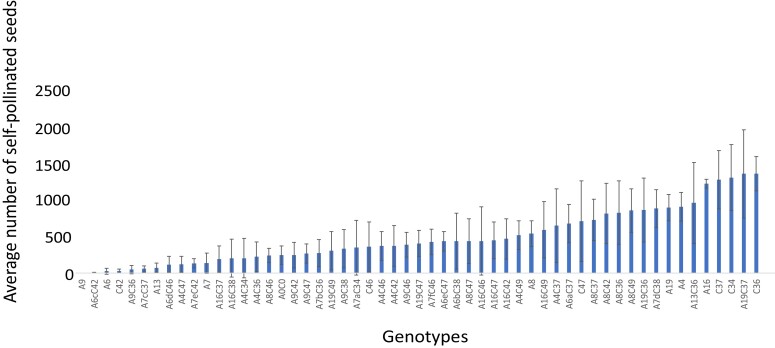
Fertility of resynthesized *B. napus* lines measured by the average number of self-pollinated seeds was compared to progenitor *B. rapa* (A4, A6, A7, A8, A9, A13, A16, and A19) and *B. oleracea* (C34, C36, C37, C42, C46, and C47) genotypes. Resynthesized lines are indicated by the parent combination in the form AnCn or AnxCn. “x” constitutes a letter from a to f representing a genetically different parent *B. rapa* of that progeny set, as *B. rapa* genotypes A6 and A7 were found to be heterozygous.

Total self-pollinated seeds per single plant in the resynthesized lines ranged from 1 to 2,067 (mean 445) (Supplementary Fig. 5a and File 6), with a mean of 45 seeds per 10 pods (range 0–148) (Supplementary Fig. 5b). Resynthesized lines also showed average pollen viability of 81% (Supplementary Fig. 6). Average number of self-pollinated seeds in resynthesized lines was higher compared to *B. rapa* and *B. oleracea* parental genotypes (Fig. 2). Average number of seeds per 10 pods and average number of self-pollinated seeds were moderately highly correlated (*r* = 0.68) (Supplementary Fig. 7), although there was no significant correlation between pollen viability and either of the seed fertility measures (Supplementary Fig. 8a and b).

### Interactions between *B. rapa* and *B. oleracea* parent genotypes affected fertility

Resynthesized *B. napus* lines were assessed in order to detect whether maternal (*B. rapa*) or paternal (*B. oleracea*) genotypes independently influence fertility (total number of self-pollinated seeds and seeds per 10 pods). The total number of self-pollinated seeds produced was significantly affected by *B. rapa* parent genotype (ANOVA, *P* = 0.000539) (Supplementary Fig. 9a) but not by *B. oleracea* parent genotype (Supplementary Fig. 9b). Neither *B. rapa* nor *B. oleracea* parent genotype independently affected seeds per 10 pods (Supplementary Fig. 10a and b). However, a significant interaction effect (1-way ANOVA, *P* = 5.97e−05, Tukey's HSD test *P* < 0.05) was observed for the combination of *B. rapa* and *B. oleracea* parent genotypes on the total number of self-pollinated seeds produced in *B. napus* resynthesized lines based on our linear model (Supplementary Table 1).

### Frequent CNVs detected in resynthesized *B. napus* lines

**Fig. 3.**
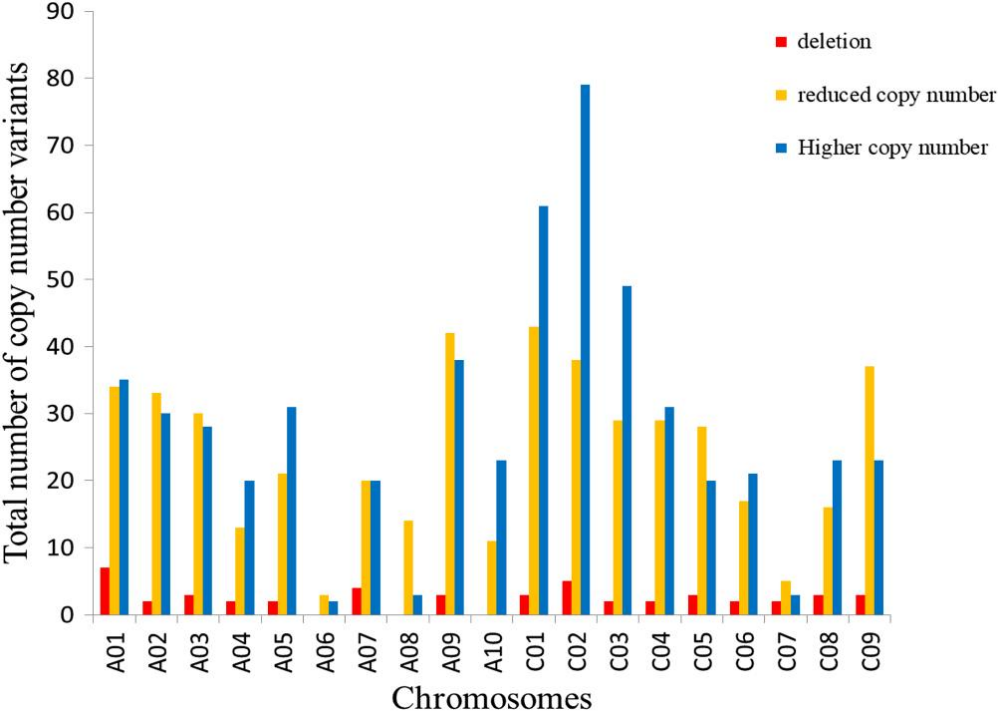
Genome-wide distribution of CNVs detected in resynthesized *Brassica napus* individuals. Deletions (zero copies of a chromosome region) are indicated in red, reduced copy (1 copy of a chromosome region) in yellow, and higher copy number (3 or more copies of a chromosome region) in blue.

Copy number variants (deletions, reduced copy numbers, and higher copy numbers) were detected at a high frequency across the A and C genomes in the resynthesized *B. napus* lines (Fig. 3). The total number of CNVs detected varied widely between resynthesized *B. napus* individuals (Supplementary Fig. 11 and File 7). No CNVs (>0.5Mb) observed in the resynthesized *B. napus* lines appeared to be inherited from their A or C genome parents.

### *B*. *rapa* and *B. oleracea* parent genotypes interact to affect genome stability (number of copy number variants) in resynthesized rapeseed lines

**Fig. 4.**
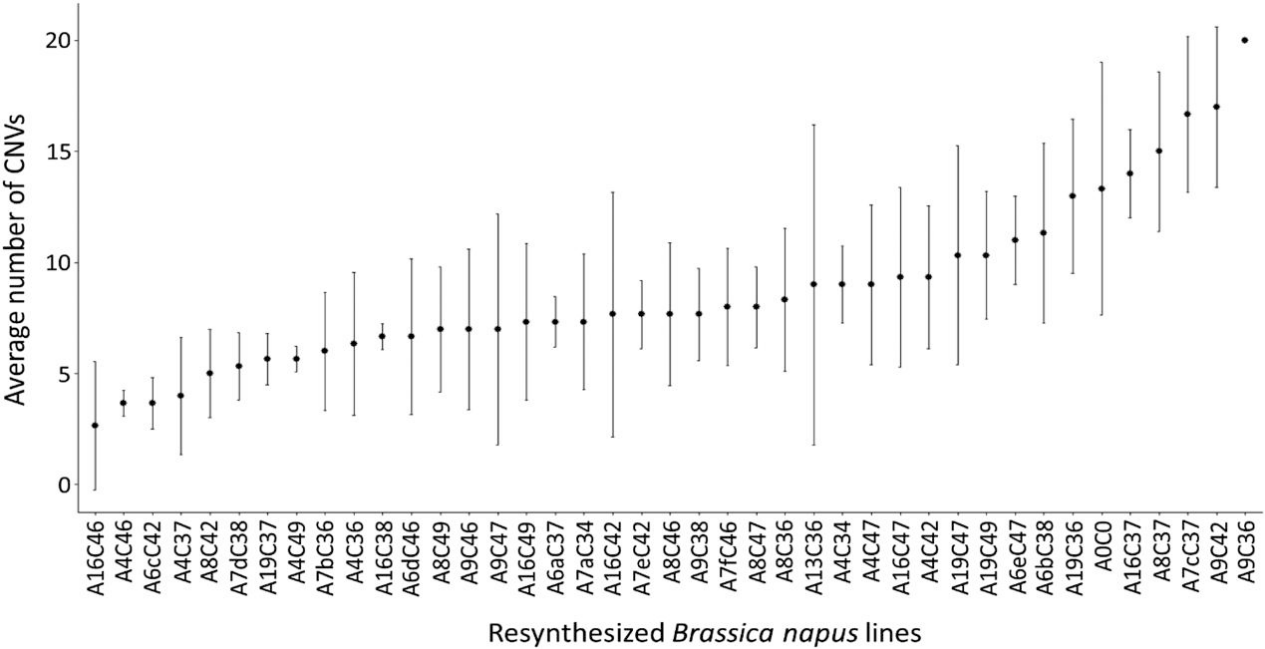
Average number of CNVs in resynthesized *B. napus* (rapeseed) lines comprising different genotype combinations (3 individuals per line). Parent genotypes of *B. rapa* are A4, A6, A7, A8, A9, A13, A16, and A19; parent genotypes of *B. oleracea* are C34, C36, C37, C38, C42, C46, C47, and C49; and synthetic rapeseed lines are indicated by the parent combination in the form AnCn or AnxCn. “x” constitutes a letter from a to f representing a genetically different parent *B. rapa* of that progeny set, as *B. rapa* genotypes A6 and A7 were found to be heterozygous. Statistical comparisons between lines can be found in Supplementary Table 2.

Although parent *B. rapa* and *B. oleracea* genotypes independently had no significant effect on the number of CNVs, the interaction between the 2 parent genotypes was significant, such that there were significant differences between resynthesized lines (ANOVA, *P* = 3.15e−06; Fig. 4; Tukey's HSD test *P* < 0.05, Supplementary Table 2). Hence, the number of CNVs detected was affected by different cross combinations of *B. rapa* and *B. oleracea* parent genotypes based on our linear model.

The relationship between genome stability (as measured by number of CNVs) and fertility was assessed in the resynthesized *B. napus* individuals. Total number of copy number variants per individual was not significantly correlated with fertility as measured by seeds per 10 pods or pollen viability in resynthesized *B. napus* individuals. A relatively weak significant relationship (Spearman rank correlation = −0.24, *P* = 0.04) was observed between total number of copy number variants per individual and total seed set per individual. This indicates that other factors apart from genome stability also contribute to fertility in resynthesized lines.

### Allelic state of resynthesized S_1_*B. napus* lines predicted by estimating CNVs

CNVs showed a relatively continuous distribution from low to high across the resynthesized *B. napus* lines. However, in order to carry out further analyses, 19 resynthesized *B. napus* lines, excluding cross combinations with heterozygous A6 and A7 parents, were classified into stable (more stable), intermediate, and unstable (less stable) using the following criteria and process. Firstly, we undertook pairwise comparisons of CNV data to determine which resynthesized lines were significantly different from each other (Tukey's HSD test *P* < 0.05, Supplementary Table 2). Two groups were established which were significantly different from each other in numbers of CNVs: these lines were classified as either putatively “stable” (low numbers of CNVs) or putatively “unstable” (high numbers of CNVs) while lines which were not significantly different from any other line were classified as “intermediate.” Based on this, resynthesized lines with average number of CNVs below 6 were classified into putatively “stable” (4 combinations), from 6 to 10 putatively “intermediate” (11 combinations), and above 10 as putatively “unstable” (4 combinations) (Table 1).

**Table 1.** Classification of resynthesized *Brassica napus* cross combinations resulting from homozygous parent *B. rapa* and *B. oleracea* genotypes into putatively “stable,” putatively “unstable,” and putatively “intermediate” by pairwise comparisons and estimation of average numbers of CNVs.

	C34 var bot dh	C36 var bot dh	C37 var bot dh	C46 var alb il	C47 var alb il
A4 var tri il	Intermediate	Intermediate	Stable	Stable	Intermediate
A16 var tri il	—	—	Unstable	Stable	Intermediate
A19 var tri il	—	Unstable	Stable	—	Intermediate
A8 var tri dh	—	Intermediate	Unstable	Intermediate	Intermediate
A9 var olei dh	—	Unstable	—	Intermediate	Intermediate
A13 var pek dh	—	Intermediate	—	—	—

Genotypes without data (no plants) are indicated with “—.” Lines are from Abel *et al.* (2005). var olei, *B. rapa* var. *oleifera*; var tri, *B. rapa* var. *trilocularis*; var bot, *B. oleracea* var. *botrytis*; var alb, *B. oleracea* var. *alboglabra*; dh, double haploid; il, inbred lines.

### Allelic variation in meiosis genes is associated with number of CNVs

Eight parent accessions of *B. rapa* (A4, A6, A7, A8, A9, A13, A16, and A19) and 5 parent accessions of *B. oleracea* (C34, C36, C37, C46, and C47) were resequenced. However, *B. rapa* A6 and A7 accessions were found to be heterozygous and were subsequently taken out of the analysis. In the next step, the allelic variation was analyzed in a list of meiosis gene homologs. A total of 3,689 SNPs in *B. rapa* meiosis genes were detected, of which 832 were nonsynonymous and no splice variants were detected (Supplementary File 7a). In *B. oleracea* meiosis genes, 2,549 SNPs were detected, of which 729 were nonsynonymous, 4 were splice variants, and 3 were stop codon gains (Supplementary File 7b). Moreover, CNVs in meiosis genes were detected by analyzing coverage. It was found that 96 of the 197 *B. rapa* meiosis gene copies predicted from the reference genome carried a deletion in at least 1 accession, out of which 2 were deleted in all 8 accessions, and 90 gene copies carried a duplication (Supplementary File 7c). In *B. oleracea*, 33 gene copies out of 193 were deleted in at least 1 accession, 1 of them in all accessions, and 57 were duplicated (Supplementary File 7d).

From these data, the allelic state of the S_1_*B. napus* resynthesized lines by combining the respective parents was inferred (Supplementary File 7e and f) from which the list of putative meiosis genes candidates was pulled (Supplementary File 8a–d). Phenotypic data for 19 cross combinations (excluding combinations with heterozygous A6 and A7 parents) which could be tested in the greenhouse were used, and total CNV counts genome-wide classified lines into putatively “stable” (4 combinations), “intermediate” (11 combinations), “unstable” (4 combinations), and “missing” (11 combinations) (Table 1). In the next step, meiosis candidate genes were selected using the following criteria (Supplementary File 8a–e): firstly, significant associations of number of CNVs with meiosis gene haplotypes after FDR correction (Supplementary File 8b and e); secondly, presence of putatively harmful mutations in meiosis gene haplotypes which fulfill criterion 1 (nonconservative missense codon, stop codon gain variants, and/or splice variants) (Supplementary File 8b and e); and thirdly, putative gene function in meiosis related to DNA or double-strand break repair, effects on meiotic crossover, or effects on homoeologous recombination (Supplementary File 8e).

Using these 3 criteria, we identified 13 putative meiosis genes from the *B. oleracea* C genome parents used to produce the resynthesized *B. napus* lines (Table 2). Of these, *RPA1C*, *MSH2*, and *RECQ4B* also showed presence of either a stop codon or a splice variant in at least 1 gene copy. *RPA1C* gene copies carried 2 nonconservative missense codons and 1 stop codon gain. *MSH2* carried 1 stop codon gain and 1 splice donor variant, while *RECQ4B* showed 4 missense codons, 1 stop codon gain variant, and 1 splice acceptor variant. Other putative candidate genes with significant CNV association with haplotypes and carrying at least 1 nonconservative missense codon were *BRCA*, *ATR*, *RAD51C*, *MLH3*, *RECQ4A*, *SDS*, *RAD51*, *BLAP75/RMI1*, *SYN1/DIF1/REC8*, and *AtGR1/COM1* (Table 2). In the *B. rapa* parents, none of the gene haplotypes fulfilled the criterion of significant (after FDR correction) association with total CNV number, which was our most important criterion for the selection of putative meiosis gene candidates. So these genes from *B. rapa* parents (Supplementary File 8c and d) were not considered as interesting meiosis gene candidates.

**Table 2.** Meiosis gene haplotypes associated with CNV frequencies in resynthesized *B. napus* derived from crosses between *B. rapa* and *B. oleracea* parents.

Candidate genes involved	Chromosome	Location of other copies	*B. oleracea* copies	*A. thaliana* homolog	Type of mutation	Significant CNV association with haplotypes after FDR correction (*P* < 0.05)
*RPA1C*	C02	C09	*Bo2g127130.1*, *Bo9g061490.1*	*AT5G45400.1*, *AT5G45400.1*	Nonconservative missense codon, stop codon gain	0.04, 0.04
*MSH2*	C06	C06, C03	*Bo6g030570.1*, *Bo3g071550.1*, *Bo6g003510.1*	*AT3G18524.1*, *AT3G18524.1*, *AT3G18524.1*	Stop codon gain, splice variant donor	*P* > 0.05, 0.03, *P* > 0.05
*RECQ4B*	C09	—	*Bo9g043460.1*	*AT1G60930.1*	Nonconservative missense codon, stop codon gain, splice acceptor variant	0.04
*AtGR1/COM1*	C04	—	*Bo4g125630.1*	*AT3G52115.1*	Nonconservative missense codon	0.03
*ATR*	C04	—	*Bo4g145640.1*	*AT5G40820.1*	Nonconservative missense codon	0.03
*BRCA*	C01	C01	*Bo1g023820.1*, *Bo3g001340.1*	*AT4G21070.1*, *AT5G01630.1*	Nonconservative missense codon	0.03, 0.04
*RAD51C*	C04	—	*Bo4g038470.1*	*AT2G45280.1*	Nonconservative missense codon	0.04
*MLH3*	C07	—	*Bo7g117460.1*	*AT4G35520.1*	Nonconservative missense codon	0.03
*RECQ4A*	C08	C08	*Bo8g059730.1*, *Bo8g109200.1*	*AT1G10930.1*, *AT1G10930.1*	Nonconservative missense codon	0.03, 0.03
*SDS*	C08	C05	*Bo8g066230.1*, *Bo8g106580.1*, *Bo5g019980.1*	*AT1G14750.1*, *AT1G14750.1*, *AT1G14750.1*	Nonconservative missense codon	0.04, 0.03, 0.03
*RAD51*	C09	C03	*Bo9g151450.1*, *Bo3g015380.1*	*AT5G20850.1*, *AT5G20850.1*	Nonconservative missense codon	0.04, 0.03
*BLAP75/RMI1*	C09	—	*Bo9g017740.1*	*AT5G63540.1*	Nonconservative missense codon	0.03
*SYN1/DIF1/REC8*	C09	—	*Bo9g177100.1*	*AT5G05490.1*	Nonconservative missense codon	0.04

### Genetic analyses of resynthesized *B. napus* lines

Analysis of the genetic background of the parental lines used in this study showed that maternal *B. rapa* var. *trilocularis* was the parent for all stable resynthesized lines (4 out of 4 considered as putatively stable), although this subspecies also contributed to intermediate (8 out of 11) and unstable lines (3 out of 4) (Table 1). Resynthesized cross combinations with C46 (*B. oleracea* var. *alboglabra*) as paternal *B. oleracea* parent were also either putatively stable or intermediate (Table 1). Although it is not possible to draw statistically significant conclusions from these results in the current study, further investigation of this association may be warranted in future.

## Discussion

In this study, we aimed to test the hypothesis that allelic variants inherited from parental genotypes of *B. rapa* and *B. oleracea* would affect meiosis in newly resynthesized rapeseed lines. To this end, we analyzed relative genome stability (as measured by copy number variants) and measured fertility using self-pollinated seed set, seeds per 10 pods, and pollen viability in a set of lines of resynthesized *B. napus* with common parent genotypes after 1 generation of self-pollination as well as screened for variants of meiosis candidate genes possibly affecting genome stability in the lines. Our results show that allelic variants inherited from both diploid *B. rapa* and *B. oleracea* parents interact to affect genome stability and fertility in resynthesized *B. napus* lines. Resynthesized rapeseed lines from different genetic backgrounds also vary significantly in both fertility and genome stability. We identified 13 putative meiosis candidate genes which were significantly associated with frequency of copy number variants and which contained putatively harmful mutations in meiosis gene haplotypes for further investigation.

Our results show a negative correlation between genome stability (CNVs) and fertility as measured by total seed set (*P* = 0.04, Spearman correlation *r* = −0.24) in resynthesized *B. napus*. In natural *B. napus*, inheritance of unbalanced translocation events in mapping populations was also associated with a fertility penalty (Osborn *et al.* 2003). Negative correlations between chromosome rearrangements and fertility have also been observed in both natural and resynthesized *B. napus* populations (Samans *et al.* 2017). These results support the present study where CNVs were significantly negatively associated with fertility (self-pollinated seed set). However, the detected correlation was low, indicating that other factors apart from genome stability are contributing to fertility in resynthesized *B. napus* lines. As well, we would predict that in translocation heterozygotes (indicated by CNVs where we see 1 or 3 copies of a chromosomal region), both size and relative A–C genome homoeology/chromosomal location of the CNV would have large effects, although we were not able to resolve this level of detail in the current study.

We observed a wide range of genotype-dependent fertility across the resynthesized *B. napus* lines, and some resynthesized lines showed higher fertility than *B. rapa* and *B. oleracea* parental genotypes in our study. Malek *et al*. (2012) detected higher fertility in synthetic *B. napus* compared to its parental *B. rapa* and *B. oleracea* genotypes in terms of the number of seeds per silique, 1000-seed weight, and seed yield per plant, in support of the hypothesis that polyploid crops (interspecific hybrids) often show higher yield levels and outperform their diploid relatives (Sattler *et al.* 2016), highlighting the heterotic potential of resynthesized *B. napus* lines (Abel *et al*. 2005). Rousseau-Gueutin *et al*. (2017) assessed fertility (number of seeds/50 pollinated flowers and number of seeds/50 pods) in both open-pollinated and manually self-pollinated resynthesized *B. napus* populations and found very low fertility compared to natural *B. napus* using both fertility measures. Rousseau-Gueutin *et al*. (2017) hypothesized that self-incompatibility alleles carried by the parental diploid species might have affected the fertility of their hybrids, since different subspecies of *B. rapa* and *B. oleracea* parents had been used to produce the resynthesized *B. napus* population. Many genotypes of *B. rapa* and *B. oleracea* are self-incompatible, a trait genetically controlled by the self-incompatibility *S*-locus (Camargo *et al.* 1997; Kimura *et al.* 2002; Kitashiba and Nasrallah 2014), which prevents self-seeds. Self-pollinated seed set varied greatly across our *B. rapa* and *B. oleracea* parents and their progeny, with possible self-incompatibility issues in a few parental genotypes used to produce the resynthesized lines (Fig. 2). One genotype, *B. rapa* A9, was most likely self-incompatible, setting no self-pollinated seed. Interestingly, none of the synthetic combinations with A9 were completely sterile, and some were highly fertile, suggesting the synthetic *B. napus* mostly overcame self-fertilization via recognition in the stigma and failed germination of pollen with the same *S*-haplotype as the parent plant. However, self-incompatibility alleles present in some parent genotypes used to produce our resynthesized lines might be responsible for low fertility in a few lines (9 genotypes produced <15 seeds). Further investigation would be needed to confirm this.

In this study, most of our resynthesized *B. napus* genotypes averaged >5 CNVs per plant across the A and C genomes. Several studies have shown that chromosomal rearrangements occur frequently in both resynthesized and natural *B. napus* (Pires *et al.* 2004; Udall *et al.* 2005; Leflon *et al.* 2006; Liu *et al.* 2006; Nicolas *et al.* 2007; Chalhoub *et al.* 2014; Guo *et al.* 2016). Homoeologous exchanges have been detected in translocated regions (deletion–duplication events) between A and C homoeologous chromosomes in both resynthesized and natural *B. napus*, where we see a gradient of decreasing translocation frequency with decreasing size of homoeologous regions between the A and C genomes (Chalhoub *et al.* 2014; Samans *et al.* 2017; Mason *et al.* 2018). This pattern was also observed in our study. Samans *et al*. (2017) analyzed 52 highly diverse *B. napus* genotypes including 32 natural and 20 synthetic *B. napus* accessions using whole genome sequencing and detected a greater number and size of genomic rearrangements in synthetic *B. napus* compared to natural accessions as well as more areas with deletions than duplications. We also found fewer deletions (5.6%) than either reduced (1 missing copy) or higher copy number (1 or 2 extra copies as predicted by the pipeline) variants. However, our inability to discriminate between 3 or 4 copies (the combined “higher copy number” category) prevents us from making conclusions about the prevalence of deletions relative to duplications. Also, in contrast to the abovementioned studies, which were all on established lines of *B. napus* or synthetic *B. napus* which had been through many generations of self-pollination, our resynthesized *B. napus* material has undergone only 1 self-pollination event (2 meioses). Hence, any novel variants which have arisen are unlikely to be “fixed” (homozygous, present in both homologous chromosomes), as the products of a single homoeologous crossover event during meiosis rarely segregate together: 2 recombinant chromatids are produced from 1 homoeologous crossover, but these are usually separated into different gametes in the first meiosis. Subsequently, selection against unbalanced translocations may “fix” these events in self-pollinated progeny, resulting in balanced duplication/deletions.

Our copy number pipeline was not robust enough to efficiently discriminate whether 3 copies of an allele or more copies (4+) were present, leading us to use a combined “higher copy number” category. Our copy number pipeline may also be overestimating reduced and higher copy number CNV calls. Failed SNP calls from the 60K *Brassica* SNP chip used possibly lowered the average copy numbers across specific regions, leading to high false positive error rates (Mason *et al.* 2017). In addition, lower signal detection may result from other types of sequence polymorphism other than CNVs, which may also result in false positives as reported by Zmieńko *et al*. (2014). Although both hybridization-based arrays and next-generation sequencing approaches used for the detection of CNVs have different limitations (Zmieńko *et al.* 2014), high-coverage sequencing likely provides a more robust method of calling CNVs (Yoon *et al.* 2009). However, obtaining sufficient read depth is still factorially more expensive than calling CNVs from array data (Mason *et al.* 2017).

We observed that different cross combinations of *B. rapa* and *B. oleracea* genotypes significantly affect genome stability (as measured by number of CNVs after self-pollination; 2 meiosis events) based on our linear model. This observation is likely due to interactions between specific allelic variants from the parent genotypes which influenced genome stability in the resynthesized lines. Hypothetically, different allelic variants present in *B. rapa* and *B. oleracea* may be present but only have an effect on meiosis after combination of these 2 genomes in a single cell. Such an effect was observed for genetic locus *PrBn* (Jenczewski *et al.* 2003), which clearly segregated allohaploid *B. napus* (2n = AC) into “high-pairing” and “low-pairing” phenotypes based on meiotic behavior (specifically frequency of A–C chromosome pairing observed at metaphase I), but which was found to have no effect on meiosis in established allopolyploid *B. napus* (2n = AACC). As well, *B. rapa* and *B. oleracea* are mesopolyploid genomes which show a triplicated genome structure relative to Brassicaceae relative *Arabidopsis* (The *Brassica rapa* Genome Sequencing Project Consortium *et al*. 2011; Liu *et al.* 2014; Parkin *et al.* 2014): although many meiosis gene copies are thought to have returned to a functional diploid state with only a single working gene copy (Lloyd *et al.* 2014), the extent of this pseudogenization and whether it is consistent across all genotypes (particularly those used in our study) is unknown. Therefore, another source of genetic variation is predicted to be the number of working meiosis gene copies across both parental genomes (*B. rapa* and *B. oleracea*), which may explain the observed genotypic interaction effects we found (meiotic effect as a sum of working meiosis gene copies in both genomes). In support of this hypothesis, Gonzalo *et al*. (2019) found functional compensation of meiotic phenotype was conferred by only a single working meiosis gene copy in either of the A or C genomes in knockout lines for *MSH4*. Additionally, Gaebelein *et al.* (2019) found significant QTL for fertility (as a proxy for meiotic stability) harboring different copies of the same meiosis gene in 2 genomic locations in a synthetic *Brassica* allohexaploid population. Gaeta *et al*. (2007) suggested that *B. napus* might have initially been unstable, but that alleles responsible for genetic control of meiosis inherited from 1 or both diploid progenitors may have been selected for over time, possibly by conferring improved seed set. In *A. arenosa*, selection of specific alleles of meiosis genes seems to be responsible for reduced crossover frequency, resulting in meiotic stability in the polyploid (Yant *et al.* 2013). Recently, allelic variants of *ASY1* and *ASY3* in particular were found to reduce multivalent frequencies and help regulate meiosis in polyploid *A. arenosa* (Morgan *et al.* 2020), and introgression of meiosis gene alleles from *A. arenosa* was found to help stabilize tetraploid *Arabidopsis lyrata* (Marburger *et al.* 2019). Similarly, selection of genetic variants at preexisting loci may have contributed to form stable meiosis in ancient polyploid *Brassica* (Lloyd *et al.* 2014).

We presented a summary of 13 putative meiosis gene candidates which show significant CNV association and presence of putatively harmful mutation in meiosis gene haplotypes as well as putative gene function in meiosis related to DNA or double-strand break repair, effects on meiotic crossover, or suppression of homoeologous recombination. Of the 13 genes, 3 are of special interest due to the presence of stop codons or splice variants in at least 1 copy: *RPA1C*, *MSH2*, and *RECQ4B*. Due to the functional redundancy of many meiosis genes within *Brassica* species (Lloyd *et al.* 2014), particularly in the 2n = AACC allopolyploids (Gonzalo *et al.* 2019; Higgins *et al.* 2021), loss-of-function gene mutations are excellent candidates for major phenotypic effects on meiosis.

Replication protein A (*RPA*) is a eukaryotic, single-stranded DNA-binding protein made up of 3 subunits *RPA1*, *RPA2*, and *RPA3* and plays important roles in almost all DNA metabolic pathways including *S*-phase genome replication, DNA recombination, and DNA excision repair (Aklilu *et al.* 2014). *RPA1C* has been shown to promote homologous recombination in early meiosis, which may relate to an as-yet unknown role in regulation of nonhomologous recombination, and interactions between *RPA1C* and *RPA1E* are primarily responsible for DNA repair in *Arabidopsis thaliana* (Aklilu *et al.* 2014; Aklilu and Culligan 2016). In rice, *RPA1C* is shown to be required for ∼79% of chiasma formation, and the *RPA* complex comprising *RPA1C* and *RPA2C* is required to promote meiotic crossovers (Li *et al.* 2013). In *B. napus*, *RPA1C* was found within the *BnaA9* QTL region responsible for the prevention of homoeologous chromosome pairing (Higgins *et al.* 2021). In the present study, we also found 1 copy of *RPA1C* on chromosome A09 as well as 2 copies of *RPA1C* on chromosomes C02 and C09 from the *B. oleracea* parent of resynthesized *B. napus.* The 2 C genome copies were both significantly associated with CNVs and fertility, and radical SNP mutations were observed in both C02 and C09 copies while a stop codon gene variant was predicted in the C02 copy. Hence, even though different copies of this gene were implicated in our study relative to the study of Higgins *et al*. (2021) (in the C genome rather than the A genome), we suggest that these gene copies are all excellent candidates for future functional validation (e.g. via characterization of knockout mutants and via complementation analysis using genetic transformation to see if knockin of this gene restores the observed phenotype).

MutS is an ATPase involved in mismatch recognition, a potentially key element for discrimination between homologous (more similar) and homoeologous (less similar) chromosome sequences during meiosis, with 4 MutS homologs identified in *Arabidopsis* (*AtMSH2*, *AtMSH3*, *AtMSH6*, and *AtMSH7*) on the basis of their sequence conservation (Emmanuel *et al.* 2006). The *MSH2* protein regulates meiotic recombination during prophase 1, thereby functioning in a pro-crossover role in regions of higher sequence diversity in *A. thaliana*. *AtMSH2* has also been shown to have an antirecombination meiotic effect in *A. thaliana* (Emmanuel *et al.* 2006). *MSH2* was found in the QTL interval underlying fertility on chromosome C3 in *Brassica* allohexaploids derived from a cross (*B. napus* × *B. carinata*) × *B. juncea* (Gaebelein *et al*. 2019). Here, we found 3 gene copies of *MSH2* in the *B. oleracea* parent genome: 2 copies on chromosome C06 and 1 copy on C03. Although 1 of the gene copies on C06 showed no significant association with CNV number, a stop codon gain variant and a splice variant donor were observed as allelic variants of this gene copy. However, the other C06 copy was not significantly associated with fertility or CNV traits, with no SNP mutations observed. Another gene copy on chromosome C03 was significantly associated with CNV and total seed set and contained a missense codon.

*RecQ* helicases are involved in the processing of DNA structures arising during replication, recombination, and repair throughout all kingdoms of life (Hartung *et al.* 2007). Seven different *RecQ* genes are present in *Arabidopsis*. Among them are 2 paralogs, *RECQ4A* and *RECQ4B*, which arose as a result of a recent duplication and which are nearly 70% identical on a protein level (Hartung *et al.* 2007; Schröpfer *et al.* 2014). In *Arabidopsis*, *RECQ4A* and *RECQ4B* have both been shown to limit crossovers (Fernandes *et al.* 2018; Serra *et al.* 2018), which may assist in reducing nonhomologous recombination frequency. However, an earlier study showed that *AtRECQ4B* is specifically required to promote but not to limit crossovers, a role which is different from all other known eukaryotic *RecQ* homologs (Hartung *et al.* 2007). de Maagd *et al.* (2020) investigated the role of tomato *RecQ4* on crossover formation in an interspecific cross between cultivated tomato and 1 of its wild relatives, and observed a 1.53-fold increase of ring bivalents, suggesting a less important role in limiting crossover compared to *Arabidopsis*. Here, we found *RECQ4B* on chromosome C09 in *B. oleracea* used to produce our resynthesized *B. napus* interspecific cross. *RECQ4B* was significantly associated with CNV numbers and showed predicted radical SNP mutations with a potential harmful effect on protein function, as observed by a stop codon gain variant, as well as a splice acceptor variant. Two copies of *RECQ4A*, which is the other paralog of *RECQ4B*, were found on C08. Both copies were also significantly associated with CNVs and seed set, with radical SNP mutations as indicated by the presence of nonconservative missense codons. Based on the literature, we would perhaps expect this gene to play a role in reducing nonhomologous crossovers via reduction of total crossover frequency, rather than in allowing discrimination between homologous and nonhomologous chromosomes, and hence to play a more minor role in meiotic stabilization in *Brassica*.

Genetic variation in meiosis genes in general may cause large effects on genome stability in different plant lineages (Addo Nyarko and Mason 2022). Although *B. napus* is not thought to have undergone detectable gene fractionation since formation (Chalhoub *et al.* 2014), knockout of 1 existing *MSH4* gene copy was shown to help prevent nonhomologous chromosome pairing in *B. napus* (Gonzalo *et al.* 2019), supporting the idea that loss of functional meiosis gene copies in mesopolyploids *B. rapa* and *B. oleracea* may also then contribute to formation of allopolyploids with higher meiotic stability. We could not identify any interesting meiosis gene candidates from the *B. rapa* parent genotypes based on our analysis, most likely due to the small numbers of genotypes in our study. We have identified putative meiosis genes present in the diploid *B. oleracea* progenitor genotypes used to produce our resynthesized *B. napus* lines, some of which were present in more than 1 copy. Meiosis gene copies have been shown to be under strict control, with most genes returning rapidly to single copies (Lloyd *et al.* 2014), presumably to avoid meiotic abnormalities caused by the retention of several gene copies following polyploidization. However, allelic variants of meiosis genes which are only present in a few copies could potentially have an impact on genome stability and/or fertility of resynthesized *B. napus*. Hence, the respective allelic variants of the putative meiosis genes identified are putatively good candidates for the variation in copy number observed in our study. *B. napus* itself appears to be too young (<10,000 years) to have undergone any major gene fractionation: almost all (if not all) A and C genome gene copies in *B. napus* are still intact (and expressed similarly) relative to progenitor *B. rapa* and *B. oleracea* subgenomes (Chalhoub *et al.* 2014). Limited subgenome differentiation or specialization of A and C genome copies has also been observed with regard to gene expression in synthetic *B. napus* (before meiosis) relative to its parent genotypes: although differences between the subgenomes exist, these appear to be mainly inherited directly from the progenitor diploids (e.g. Bird *et al.* 2021; reviewed by Katche and Mason 2023). However, *B. rapa* and *B. oleracea* are themselves mesopolyploids, and not all meiosis genes have been reduced to single copy in these species (Lloyd *et al.* 2014). Gene copies within these mesopolyploid diploid genomes also show major differentiation in terms of gene expression and function, with clear differences between subgenomes (The *Brassica rapa* Genome Sequencing Project Consortium *et al*. 2011; Parkin *et al.* 2014; Cheng *et al.* 2016). Hence, *B. rapa* and *B. oleracea* may contain allelic variants including loss-of-function mutations which could conceivably affect meiosis in resynthesized *B. napus*.

Our results show that some resynthesized lines are more genomically stable and fertile than others and suggest that allelic variation present in both of the diploid parents interacts to affect the chance of chromosome rearrangement and CNV events, but that the presence of such events may not always be detrimental to fertility in resynthesized *B. napus* lines. Our study suggests meiotic stability in *B. napus* arose via selection of allelic variants from its diploid progenitor species and provides information that will be useful for breeders aiming to use resynthesized lines in breeding programs. The production of genomically stable resynthesized *B. napus* lines might be useful in the future as a germplasm resource to broaden the limited genetic diversity of established *B. napus* cultivars or for hybrid breeding (Abel *et al.* 2005).

## Supplementary Material

jkad136_Supplementary_Data

## Data Availability

The data that support the findings of this study are available in the supplementary material of this article. Sequencing data generated in this project are available under BioProject accession code PRJNA724876 from the National Center for Biotechnology Information (NCBI). Supplemental material available at G3 online.
