# Supplementary material for: Genetic factors inherited from both diploid parents interact to affect genome stability and fertility in resynthesized allotetraploid Brassica napus
Source: G3 (Bethesda). 2023 Jun 14;13(8):jkad136. doi: 10.1093/g3journal/jkad136 (PMC10411605; doi:10.1093/g3journal/jkad136)
Supplement: jkad136_Supplementary_Data [file jkad136_supplementary_data.zip › Supplemental_Figures_G3-2023-404246.pdf]

Supplementary information

Genetic factors inherited from both diploid parents interact to affect genome stability and fertility in resynthesized allotetraploid *B. napus*

Elizabeth Ihien Katche[1,2], Antje Schierholt[3], Sarah-Veronica Schiessl[2,4], Fei He[1], Zhenling Lv[1,2], Jacqueline Batley[5], Heiko C. Becker[3], Annaliese S. Mason[1,2 *]

[1] Plant Breeding Department, The University of Bonn, Katzenburgweg 5, 53115 Bonn, Germany

[2] Department of Plant Breeding, Justus Liebig University, Heinrich-Buff-Ring 26-32, Giessen 35392, Germany

[3] Department of Plant Breeding Methodology, Georg-August University Göttingen, Carl-Sprengel-Weg 1, Göttingen 37073, Germany

[4] Department of Botany and Molecular Evolution, Senckenberg Research Institute and Natural History Museum Frankfurt, Senckenberganlage 25, D-60325 Frankfurt am Main, Germany

[5] School of Biological Sciences, The University of Western Australia (M084), 35 Stirling Highway, 6009 Perth Australia

* Corresponding author; annaliese.mason@uni-bonn.de

https://orcid.org/0000-0003-2701-7964

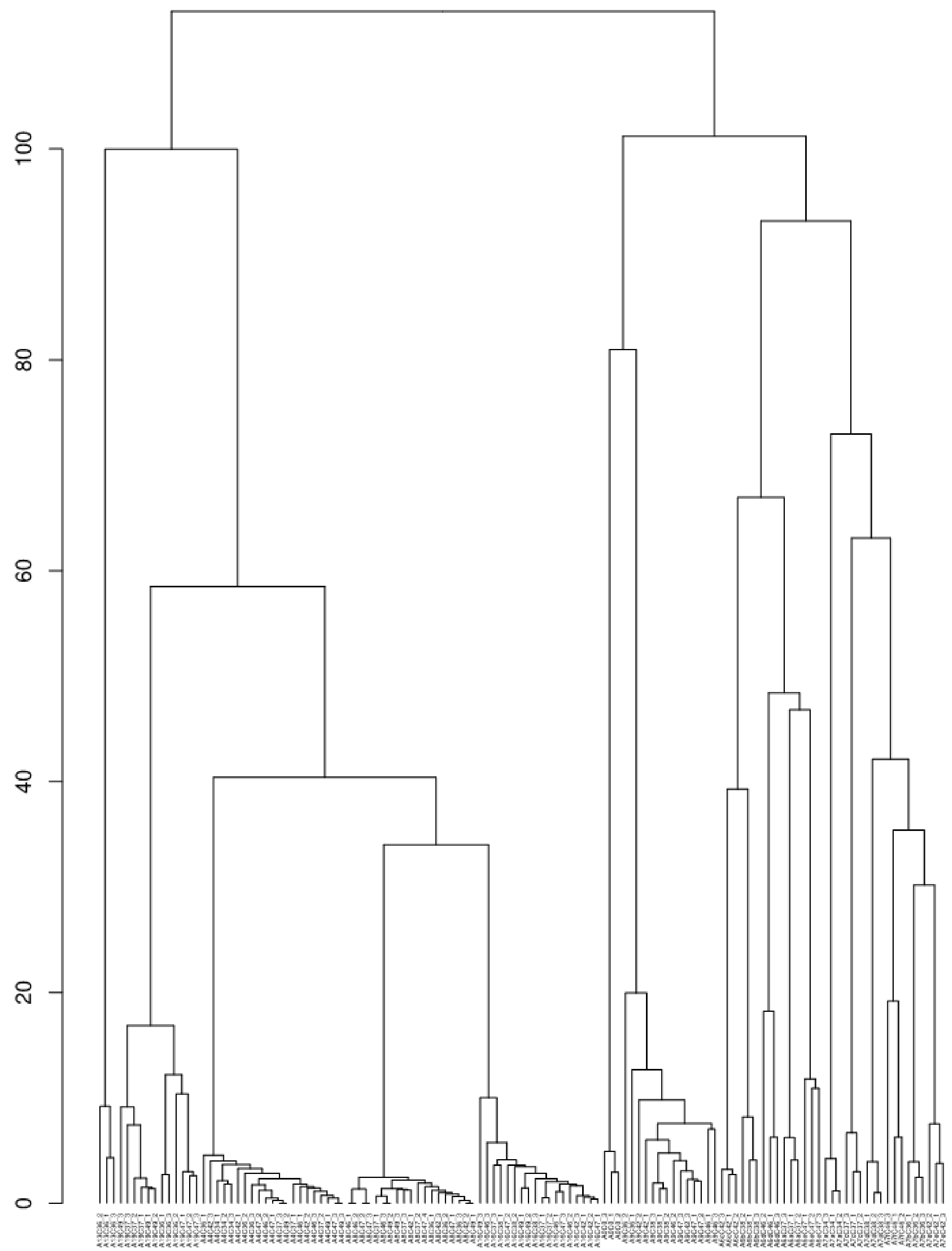

Supplementary Figure S1. Genetic distances between the A genomes of resynthesized *B. napus* lines. Although all individuals were allelically homozygous, progeny sets derived from parent *B. rapa* genotypes A6 and A7 showed divergent inheritance of parental alleles in the A genome, indicating that these parent genotypes A6 and A7 were heterozygous at the time of hybridization with *B. oleracea*.

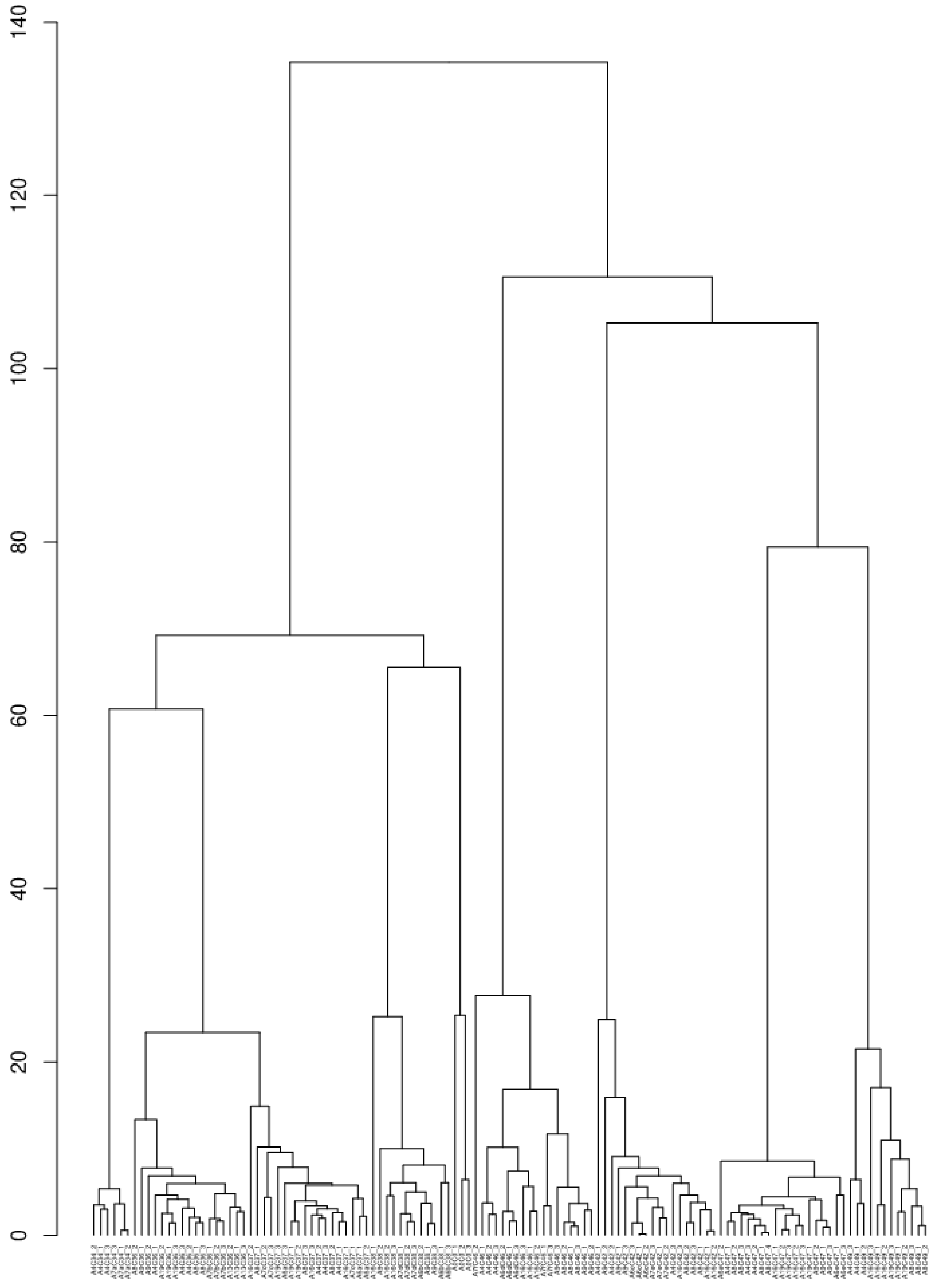

Supplementary Figure S2. Genetic distances between the C genomes of resynthesized *Brassica napus* lines. All individuals derived from the same *B. oleracea* parental cross combination showed the same genotype, indicating that the *B. oleracea* parents were homozygous as expected.

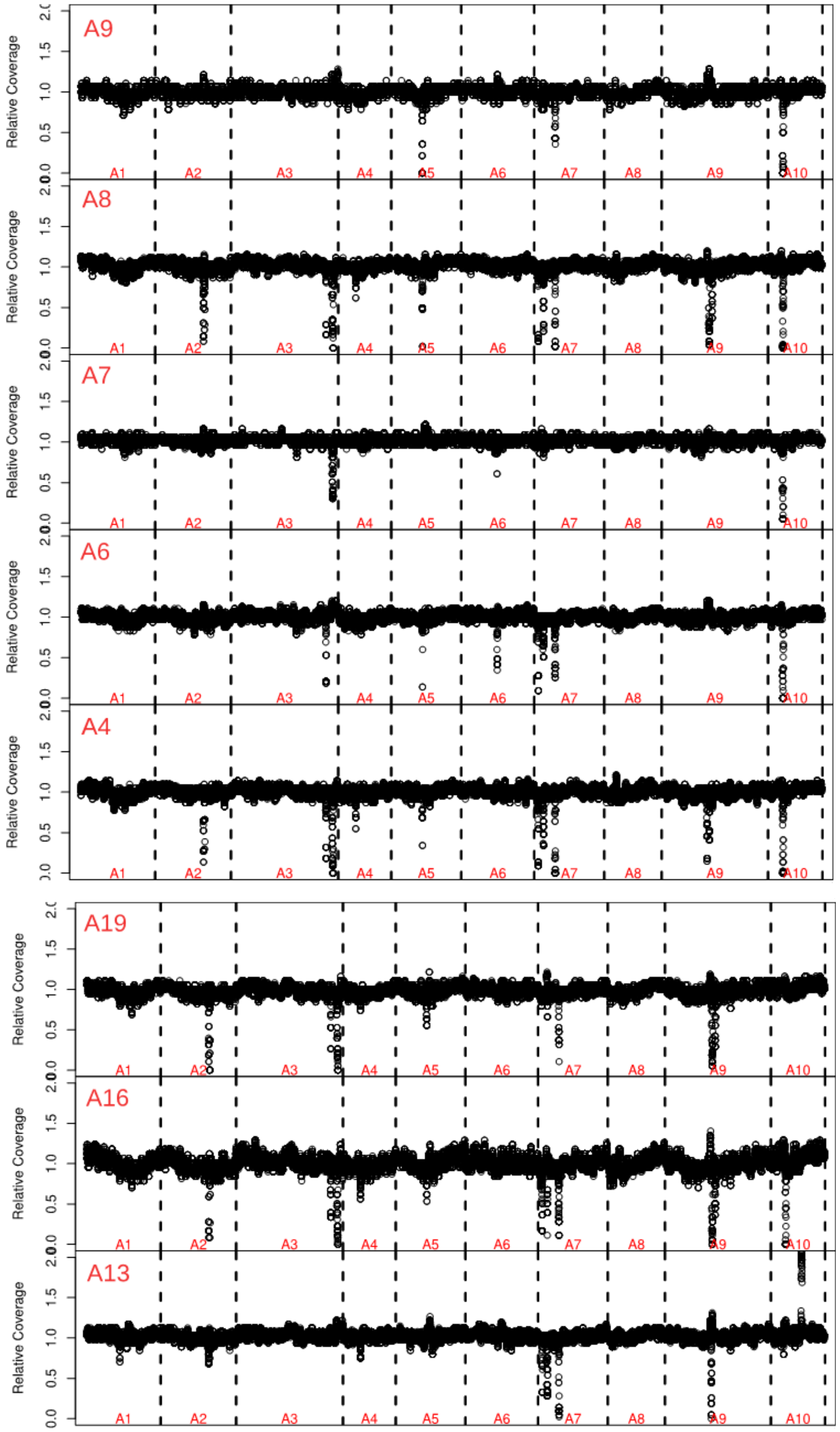

Supplementary Figure S3. Relative read coverage of sequenced *Brassica rapa* parent genotypes (A4, A6, A7, A8, A9, A13, A16 and A19) calculated for a median depth of 40 genes showing regions of the chromosomes with expected copies as well as copy number variation.

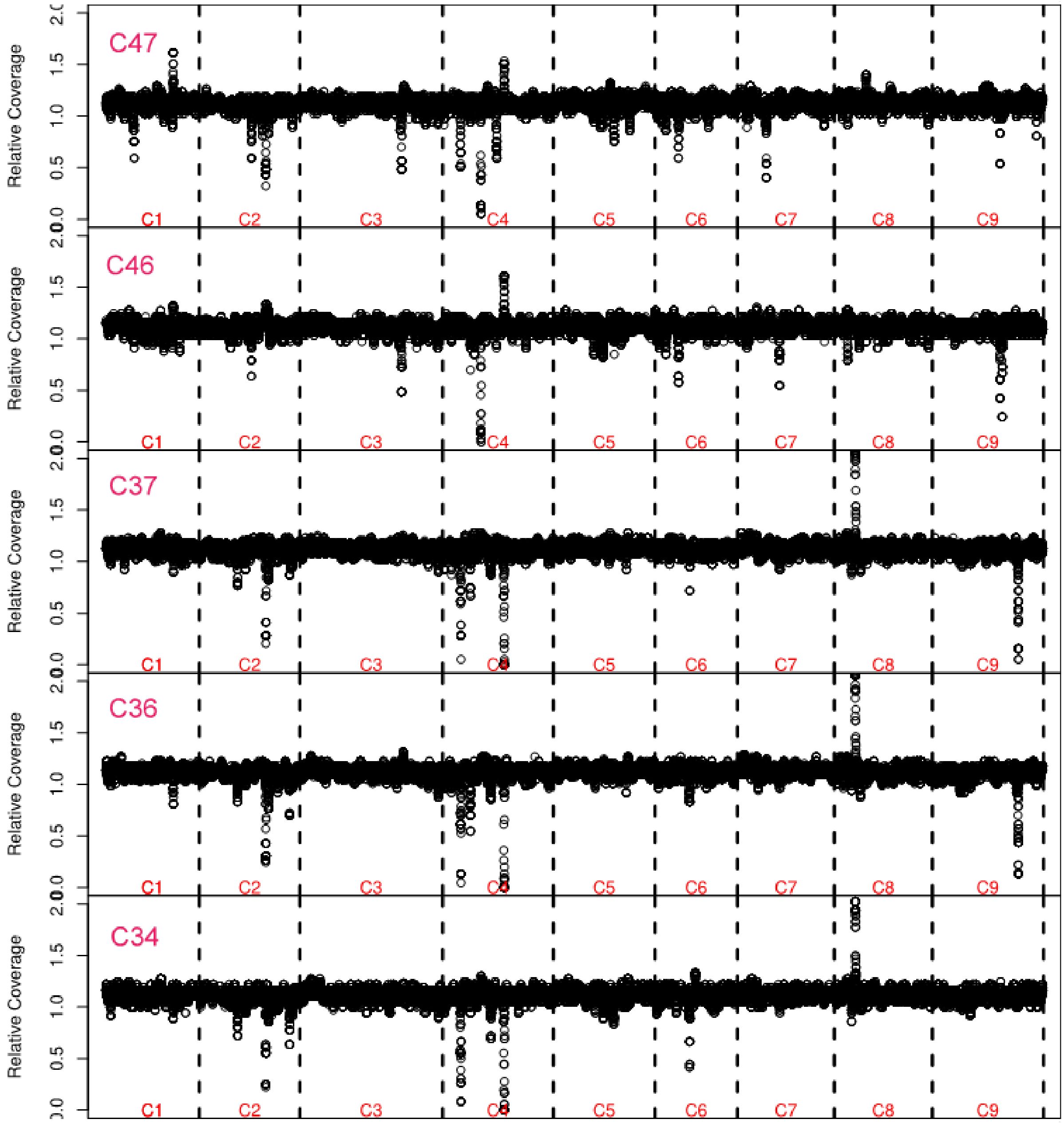

Supplementary Figure S4. Relative read coverage of sequenced *Brassica oleracea* parent genotypes (C34, C36, C37, C46, and C47) calculated for a median depth of 40 genes showing regions of the chromosomes with expected copies as well as copy number variation.

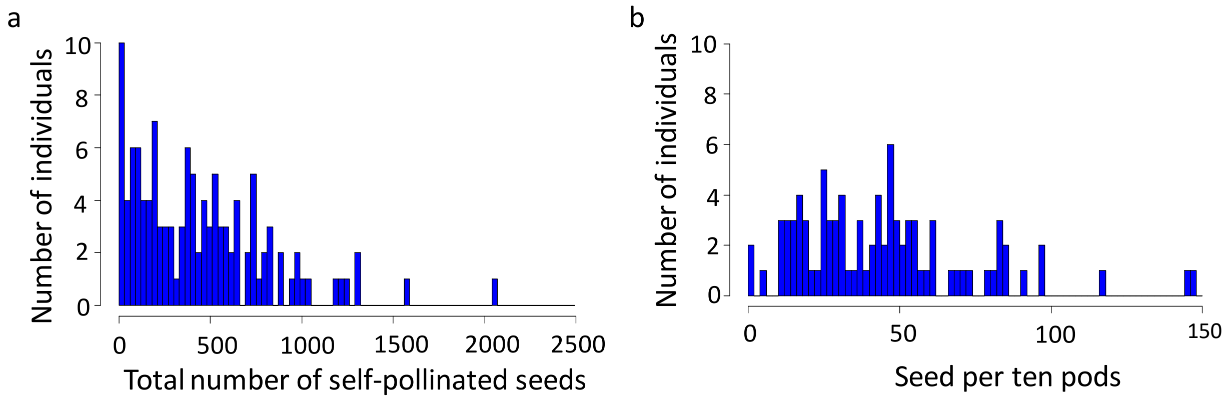

Supplementary Figure S5. Fertility of resynthesized *Brassica napus* lines was measured by the total number of self-pollinated seeds produced, as well as the number of seeds per ten pods **a)** total number of self pollinated seeds produced in resynthesized lines and **b)** number of seeds per ten pods in resynthesized lines.

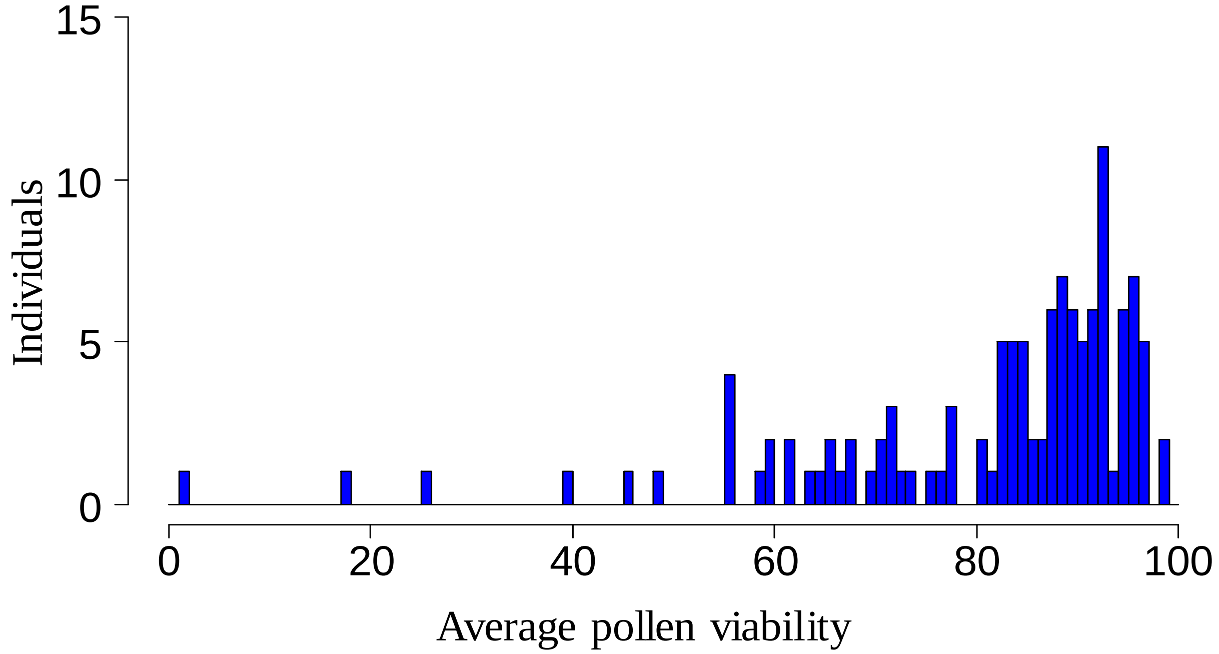

Supplementary Figure S6. Percentage pollen viability across individuals in resynthesized *Brassica napus* lines.

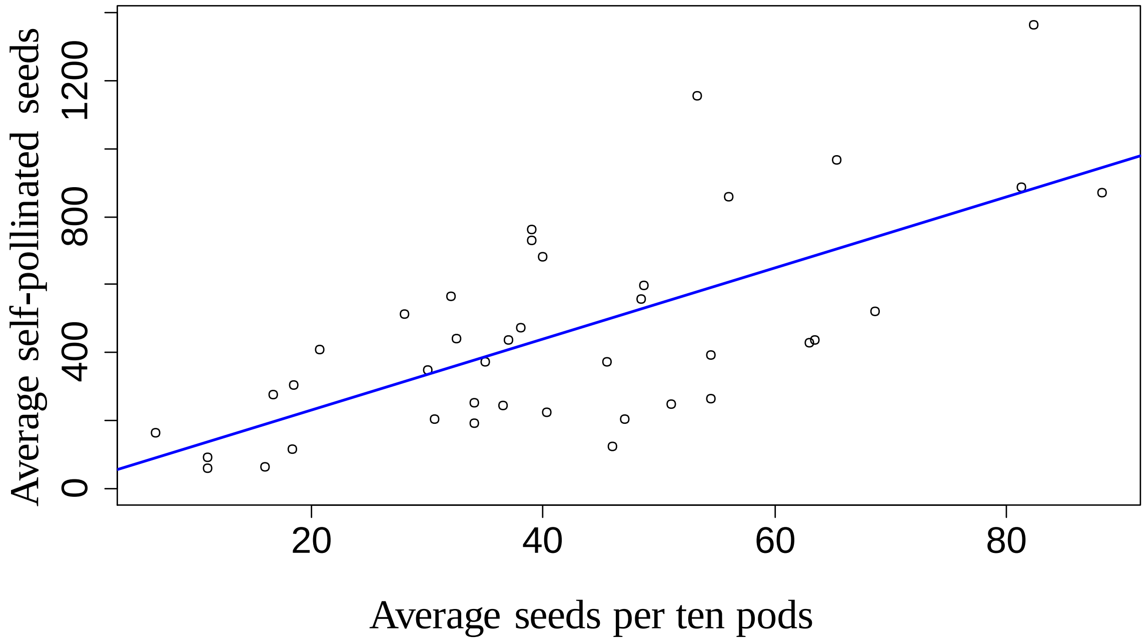

Supplementary Figure S7. Moderate positive correlation between average number of seeds per ten pods and average self-pollinated seeds in resynthesized *Brassica napus* individuals (Spearman rank correlation, $p < 0.0001$, $r = 0.68$).

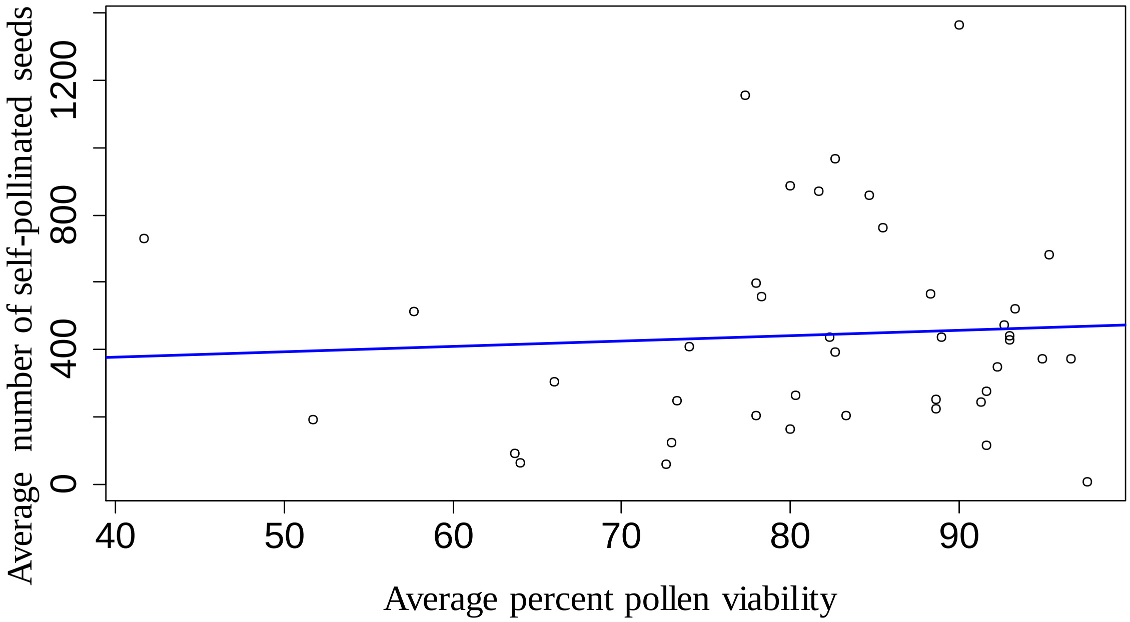

Supplementary Figure S8a. Correlation between average percent pollen viability and average self-pollinated seeds in resynthesized *Brassica napus* individuals (Spearman rank correlation, not significant, r = 0.06).

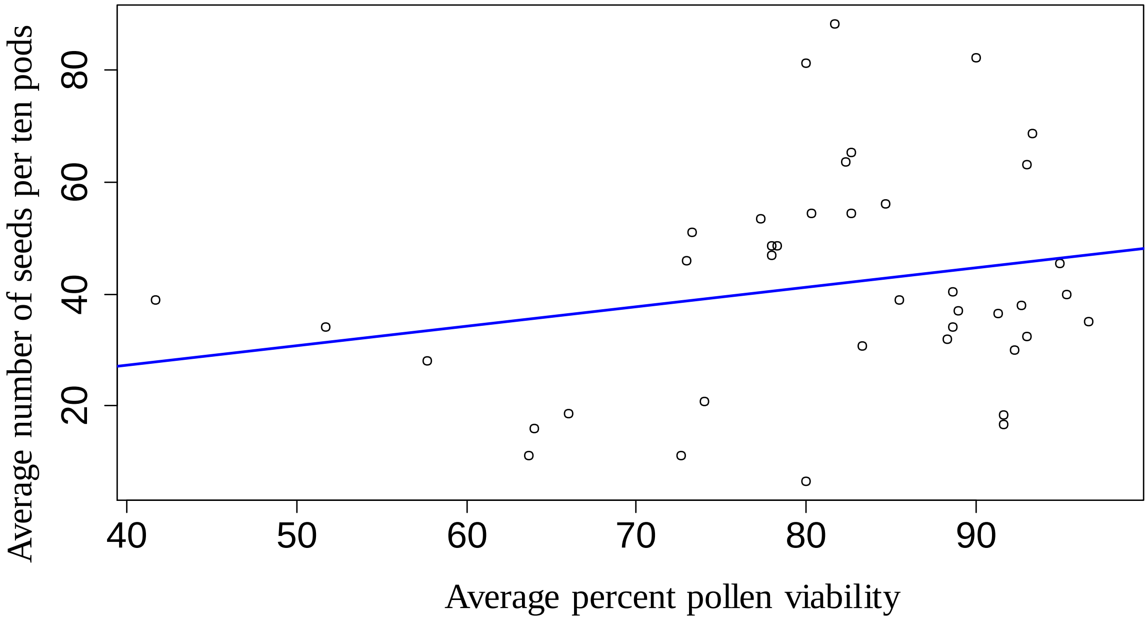

Supplementary Figure S8b. Correlation between average percent pollen viability and average seeds per ten pods in resynthesized *Brassica napus* individuals (Spearman rank correlation, not significant, r = 0.22).

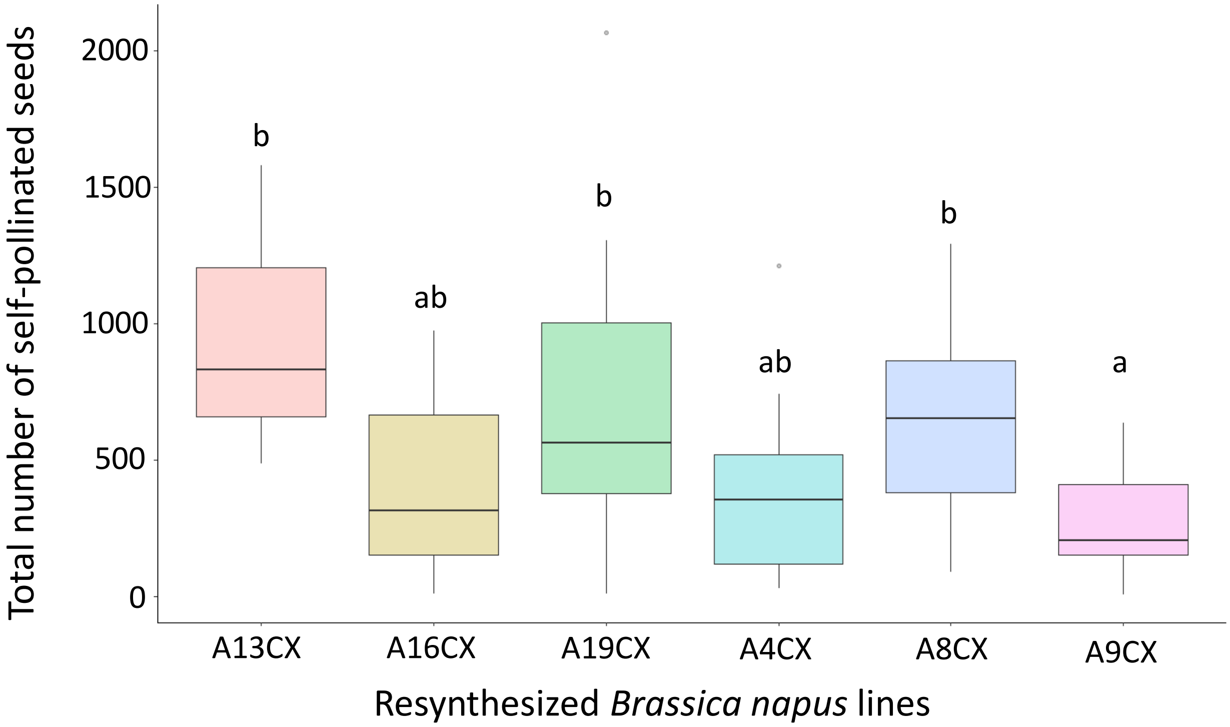

Supplementary figure S9a. *Brassica rapa* maternal genotype significantly affected the total number of self-pollinated seeds produced (ANOVA, $p = 0.000539$, Tukey's HSD, $p < 0.05$) in resynthesized *B. napus* lines produced from different combinations of *B. rapa* crossed with *B. oleracea* genotypes represented by "CX". Letters "ab" on boxplots represent non-significant differences while "a" and b represent significant differences between genotypes based on a Tukey's Honest Significant Differences test.

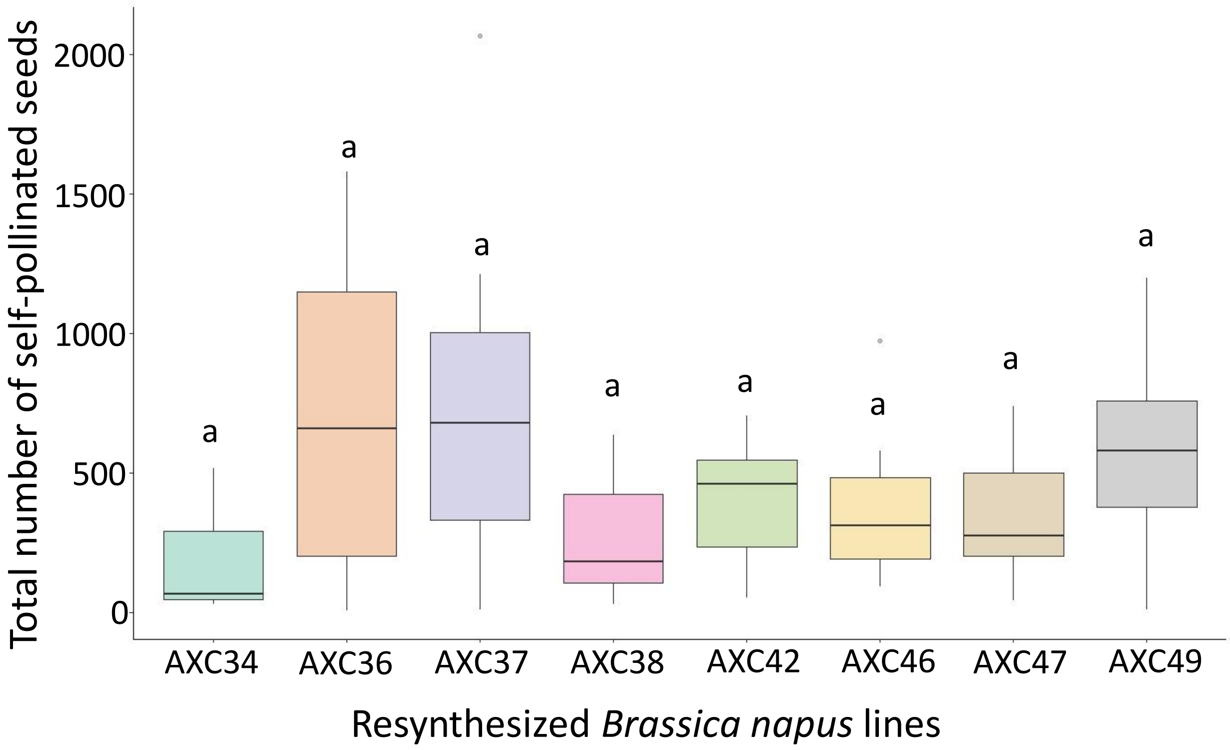

Supplementary Figure S9b. *Brassica oleracea* paternal genotypes show no significant association with total number of self-pollinated seeds (ANOVA, p= 0.068) in resynthesized *B. napus* lines produced from different *B. rapa* genotypes represented by "AX" crossed with *B. oleracea* genotypes. Letters "a" on boxplots represent no significant association between genotypes.

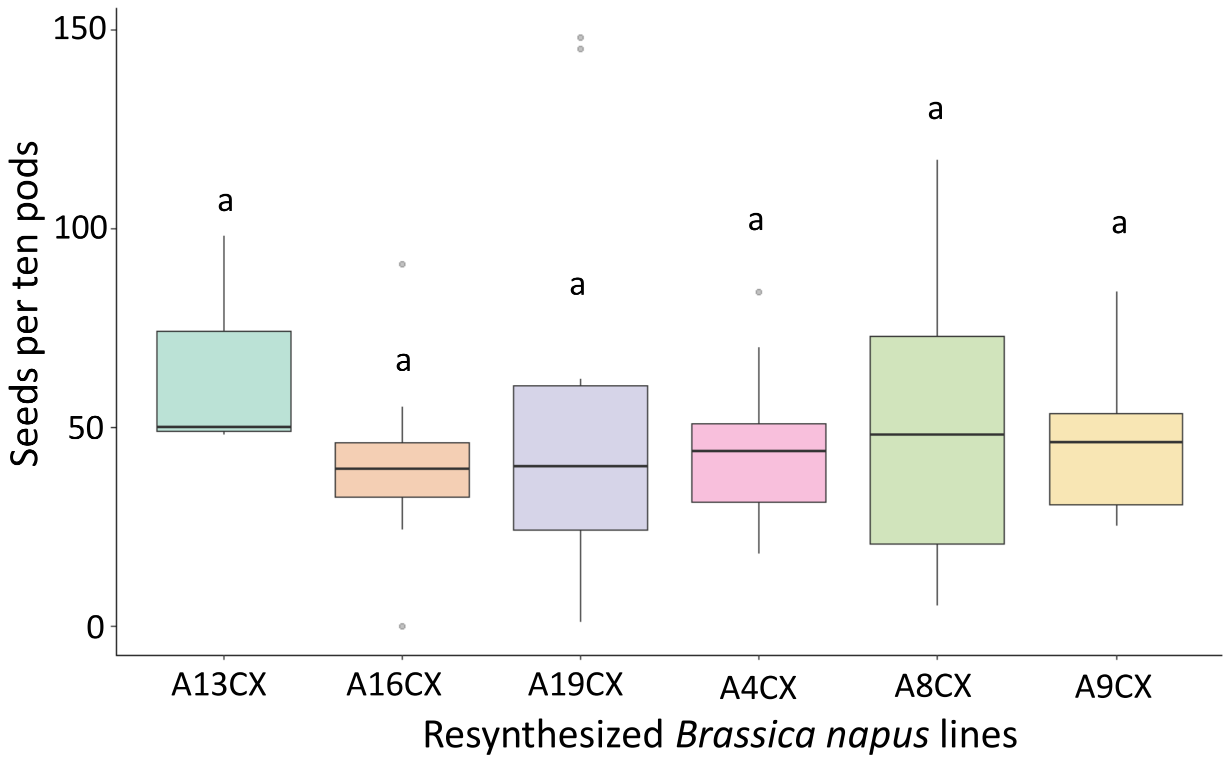

Supplementary Figure S10a. *Brassica rapa* maternal genotype showed no significant effect on the number of seeds per ten pods (ANOVA, p = 0.658) in resynthesized *B. napus* lines produced from different combinations of *B. rapa* genotypes crossed with different *B. oleracea* genotypes represented by "CX". Letter "a" on boxplots indicates no significant differences between genotypes.

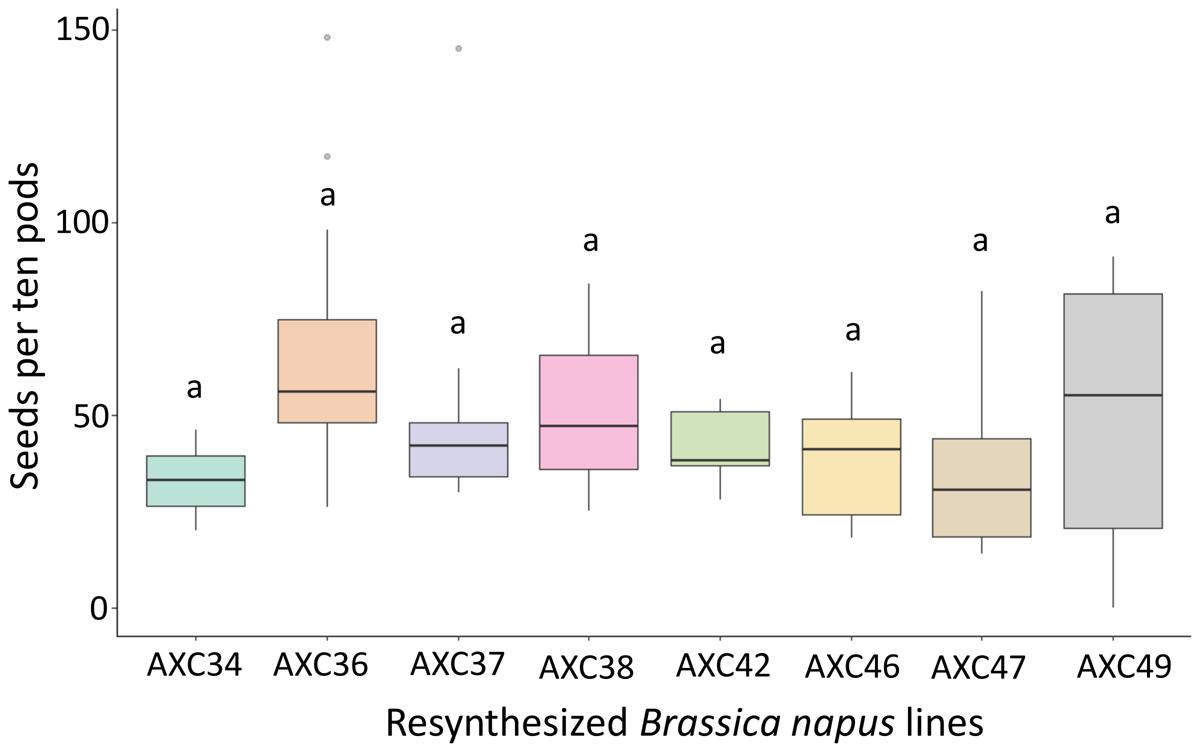

Supplementary Figure S10b. *Brassica oleracea* paternal genotype showed no significant effect on the number of seeds per ten pods (ANOVA, p = 0.0982) in resynthesized *B. napus* lines produced from different combinations of *B. rapa* represented by "AX" crossed with *B. oleracea* genotypes. Letter "a" on boxplots represents no significant differences between genotypes.

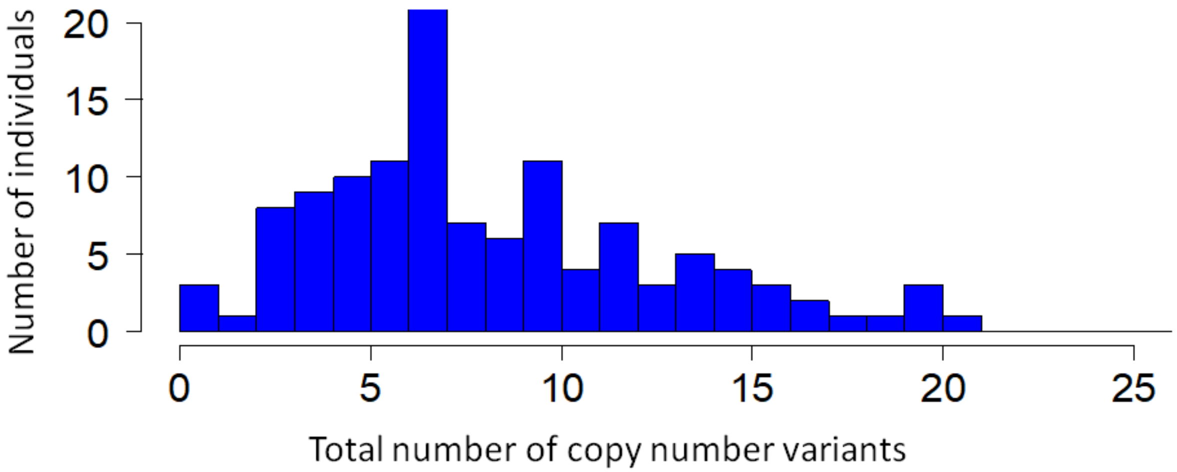

Supplementary Figure S11. Number of copy number variants present varied widely between different resynthesized *Brassica napus* individuals.

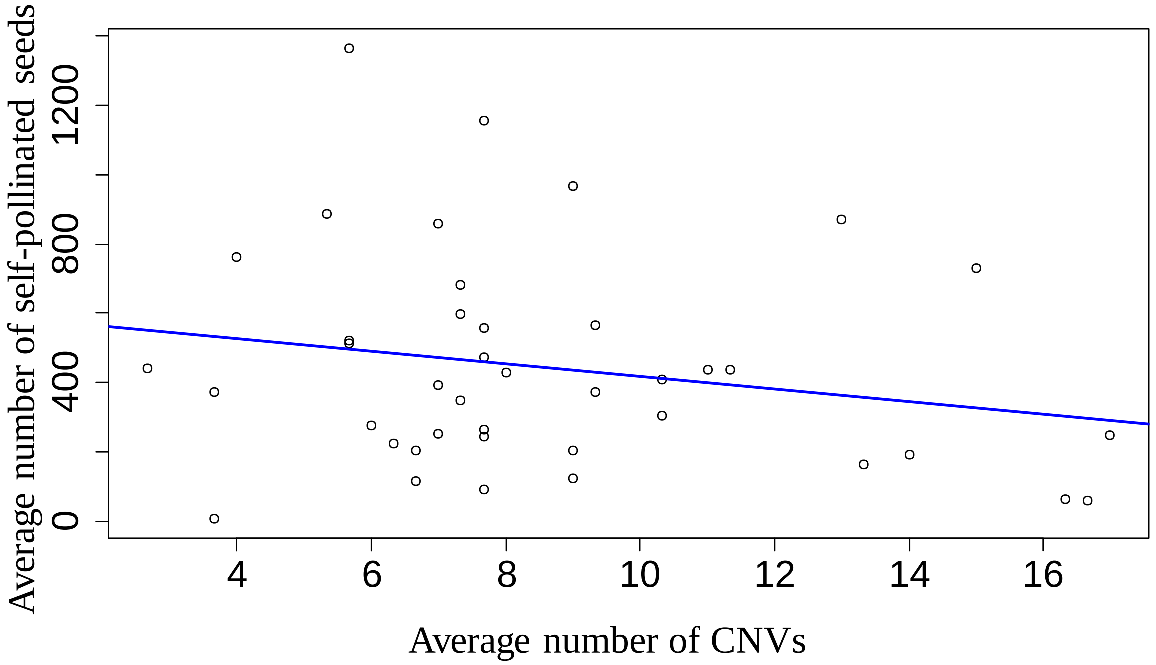

Supplementary Figure S12. Negative correlation between average number of self-pollinated seeds and average number of copy number variants in 41 resynthesized *Brassica napus* lines (Spearman rank correlation p = 0.04, r = -0.2).

.
