# Supplementary material for: Genetic factors inherited from both diploid parents interact to affect genome stability and fertility in resynthesized allotetraploid Brassica napus
Source: G3 (Bethesda). 2023 Jun 14;13(8):jkad136. doi: 10.1093/g3journal/jkad136 (PMC10411605; doi:10.1093/g3journal/jkad136)
Supplement: jkad136_Supplementary_Data [file jkad136_supplementary_data.zip › Supplemental_Material_Legends_G3-2023-404246.docx]

**Supplemental Material**

**Supplementary Figures**

**Figure S1** Genetic distances between the A genomes of resynthesized *B.* *napus* lines. Although all individuals were allelically homozygous, progeny sets derived from parent *B. rapa* genotypes A6 and A7 showed divergent inheritance of parental alleles in the A genome, indicating that these parent genotypes A6 and A7 were heterozygous at the time of hybridization with *B. oleracea*.

**Figure S2** Genetic distances between the C genomes of resynthesized *Brassica* *napus* lines. All individuals derived from the same *B. oleracea* parental cross combination showed the same genotype, indicating that the *B. oleracea* parents were homozygous as expected.

**Figure S3** Relative read coverage of sequenced *Brassica rapa* parent genotypes (A4, A6, A7, A8, A9, A13, A16 and A19) calculated for a median depth of 40 genes showing regions of the chromosomes with expected copies as well as copy number variation

**Figure S4** Relative read coverage of sequenced *Brassica oleracea* parent genotypes (C34, C36, C37, C46, and C47) calculated for a median depth of 40 genes showing regions of the chromosomes with expected copies as well as copy number variation

**Figure S5** Fertility of resynthesized *Brassica napus* lines was measured by the total number of self-pollinated seeds produced, as well as the number of seeds per ten pods a) total number of self pollinated seeds produced in resynthesized lines and b) number of seeds per ten pods in resynthesized lines

**Figure S6** Percentage pollen viability across individuals in resynthesized *Brassica napus* lines

**Figure S7** Moderate positive correlation between average number of seeds per ten pods and average self-pollinated seeds in resynthesised *Brassica napus* (Spearman rank correlation, p < 0.0001, r = 0.68)

**Figure S8a** Correlation between average percent pollen viability and average self-pollinated seed set in resynthesised *Brassica napus* (Spearman rank correlation, not significant, r = 0.06)

**Figure S8b** Correlation between average percent pollen viability and average seeds per ten pods in resynthesised *Brassica napus* (Spearman rank correlation, not significant, r = 0.22)

**Figure S9a.** *Brassica rapa* maternal genotype significantly affected the total number of self-pollinated seeds produced (ANOVA, p = 0.000539, Tukey’s HSD, p < 0.05) in resynthesized *B. napus* lines produced from different combinations of *B. rapa* crossed with *B. oleracea* genotypes represented by “CX”. Letters “ab” on boxplots represent no significant differences while “a” and b represent significant differences between genotypes based on Tukey’s Honest Significant Differences test.

**Figure S9b.** *Brassica oleracea* paternal genotypes showed no significant association with total number of self-pollinated seeds (ANOVA, p= 0.068) in resynthesized *B. napus* lines produced from different *B. rapa* genotypes represented by “AX” crossed with *B. oleracea* genotypes. Letters “a” on boxplots indicate no significant differences between genotypes.

**Figure S10a.** *Brassica rapa* maternal genotype showed no significant effect on the number of seeds per ten pods (ANOVA, p = 0.658) in resynthesized *B. napus* lines produced from different combinations of *B. rapa* genotypes crossed with different *B. oleracea* genotypes represented by “CX”. Letters “a” on boxplots indicate no significant differences between genotypes.

**Figure S10b.** *Brassica oleracea* paternal genotype showed no significant effect on the number of seeds per ten pods (ANOVA, p = 0.0982) in resynthesized *B. napus* lines produced from different combinations of *B. rapa* represented by “AX” crossed with *B. oleracea* genotypes. Letters “a” on boxplots indicate no significant differences between genotypes.

**Figure S11** Number of copy number variants present varied widely between different synthetic *Brassica napus* individuals.

**Figure S12** Negative correlation between average number of self-pollinated seeds and average number of copy number variants in 41 resynthesized *Brassica napus* lines (Spearman rank correlation p = 0.04, r = -0.2)

**Supplementary Tables**

**Table S1**: Pairwise comparison (Tukey's Honest Significant Differences) in total number of self-pollinated seeds between different resynthesized *Brassica napus* genotypes produced showing adjusted p-values. n.s. = not significant

**Table S2**: Pairwise comparisons (Tukey's Honest Significant Differences) in copy number variation between different resynthesized *Brassica napus* genotypes produced showing adjusted p-values. n.s. = not significant

**Supplementary Files**

**File S1** Description of S_1_ resynthesized *Brassica napus* lines

**File S2** SNP genotyping data for all resynthesized *Brassica napus* S_1_ individual lines across the A and C genomes after quality control

**File S3** LogR ratio values of S_1_ resynthesized *Brassica napus* lines for chromosomes A01 – A10, C1 and C2

**File S4** LogR ratio values of S­_1_­ resynthesized *Brassica napus* lines for chromosomes C3 – C9

**File S5** Meiosis gene positions determined by BLAST in *Brassica rapa* and *B. oleracea* genotypes

**File S6** Phenotype information for all resynthesized *Brassica napus* S_1_ lines

**File S7** SNP variation, copy number variation and meiosis gene positions in *Brassica rapa* and *B. oleracea* parents and in resynthesized *B. napus* lines

**File S8** List of meiosis gene candidates in *Brassica rapa* and *B. oleracea* parents and putative effect of mutations (p-values before and after FDR correction) and selection of meiosis genes of interest in *B. oleracea*
